# Characterization of oral and gut microbiome temporal variability in hospitalized cancer patients

**DOI:** 10.1186/s13073-017-0409-1

**Published:** 2017-02-28

**Authors:** Jessica R. Galloway-Peña, Daniel P. Smith, Pranoti Sahasrabhojane, W. Duncan Wadsworth, Bryan M. Fellman, Nadim J. Ajami, Elizabeth J. Shpall, Naval Daver, Michele Guindani, Joseph F. Petrosino, Dimitrios P. Kontoyiannis, Samuel A. Shelburne

**Affiliations:** 1 Department of Infectious Disease, Infection Control and Employee Health, MD Anderson Cancer Center, Houston, TX 77030 USA; 2 Department of Biostatistics, MD Anderson Cancer Center, Houston, TX 77030 USA; 3 Department of Leukemia, MD Anderson Cancer Center, Houston, TX 77030 USA; 4 Department of Genomic Medicine, MD Anderson Cancer Center, Houston, TX 77030 USA; 5 Department of Stem Cell Transplantation, MD Anderson Cancer Center, Houston, TX 77030 USA; 6 The Alkek Center for Metagenomics and Microbiome Research, Department of Molecular Virology and Microbiology, Baylor College of Medicine, Houston, TX 77030 USA; 7 Department of Statistics, Rice University, Houston, TX 77005 USA; 8 Department of Statistics, University of California, Irvine, CA 92697 USA

**Keywords:** Microbiome, Temporal variability, Leukemia, Chemotherapy, Antibiotics

## Abstract

**Background:**

Understanding longitudinal variability of the microbiome in ill patients is critical to moving microbiome-based measurements and therapeutics into clinical practice. However, the vast majority of data regarding microbiome stability are derived from healthy subjects. Herein, we sought to determine intra-patient temporal microbiota variability, the factors driving such variability, and its clinical impact in an extensive longitudinal cohort of hospitalized cancer patients during chemotherapy.

**Methods:**

The stool (*n* = 365) and oral (*n* = 483) samples of 59 patients with acute myeloid leukemia (AML) undergoing induction chemotherapy (IC) were sampled from initiation of chemotherapy until neutrophil recovery. Microbiome characterization was performed via analysis of 16S rRNA gene sequencing. Temporal variability was determined using coefficients of variation (CV) of the Shannon diversity index (SDI) and unweighted and weighted UniFrac distances per patient, per site. Measurements of intra-patient temporal variability and patient stability categories were analyzed for their correlations with genera abundances. Groups of patients were analyzed to determine if patients with adverse outcomes had significantly different levels of microbiome temporal variability. Potential clinical drivers of microbiome temporal instability were determined using multivariable regression analyses.

**Results:**

Our cohort evidenced a high degree of intra-patient temporal instability of stool and oral microbial diversity based on SDI CV. We identified statistically significant differences in the relative abundance of multiple taxa amongst individuals with different levels of microbiota temporal stability. Increased intra-patient temporal variability of the oral SDI was correlated with increased risk of infection during IC (*P =* 0.02), and higher stool SDI CVs were correlated with increased risk of infection 90 days post-IC (*P =* 0.04). Total days on antibiotics was significantly associated with increased temporal variability of both oral microbial diversity (*P =* 0.03) and community structure (*P =* 0.002).

**Conclusions:**

These data quantify the longitudinal variability of the oral and gut microbiota in AML patients, show that increased variability was correlated with adverse clinical outcomes, and offer the possibility of using stabilizing taxa as a method of focused microbiome repletion. Furthermore, these results support the importance of longitudinal microbiome sampling and analyses, rather than one time measurements, in research and future clinical practice.

**Electronic supplementary material:**

The online version of this article (doi:10.1186/s13073-017-0409-1) contains supplementary material, which is available to authorized users.

## Background

There is an increasing appreciation for the role the human microbiome plays in many aspects of human physiology, health, and disease. Several studies of healthy human cohorts have found that although each person has a relatively distinct gastrointestinal microbiome signature, a healthy individual’s microbiome remains relatively stable over time [1–4]. Although several factors, such as diet, drive normal levels of day-to-day microbiota variability, it appears that a steady-state equilibrium both ecologically and functionally is required for health. In contrast, acute perturbations of an individual’s microbiome stability within a temporal context can lead to an unhealthy status [5, 6]. Considering that one of the principal aims of the microbiome research community is to use the microbiome as either an indicator for morbidity or to improve human health, an enhanced understanding of the kinetics and taxonomic characterization of microbiome stability in acutely ill patients is of paramount importance [7–11].

Although several studies have been done in healthy subjects, relatively scant data are available as to the stability, resilience, and temporal dynamics of the gastrointestinal microbiome in acutely ill patients [1, 3, 4, 12–16]. Many of the previous investigations examining temporal variability of the microbiome using healthy participants have been limited by small numbers of volunteers [4, 12, 13], short periods of longitudinal sampling [2, 3, 16], or by being focused on only one site of collection [1, 4, 14, 17]. On the other hand, the limited number of temporal variability studies among ill patients have typically been in cohorts with chronic ailments such as atopic dermatitis or colitis [18–20]. A study of stool samples from 14 patients under intensive care described rapid shifts in microbiome composition to ultra-low diversity communities comprised of four or less taxa as a result of aggressive antibiotic treatment and other intensive care medication stresses, such as opioids [11]. Similar dramatic changes in the microbiome were also observed in patients undergoing hematopoietic stem cell transplant, where increased microbial chaos early after transplant is thought to be a potential risk factor for subsequent graft versus host disease [21, 22]. However, quantitative measurements of longitudinal microbial variability among ill patients and an analysis of factors affecting microbiome temporal stability are lacking [21, 23–25]. Moreover, despite many reports associating low microbial diversity with different illnesses, most studies associate only one-time microbiome measurements with subsequent clinical outcomes, which could be potentially problematic in settings of significant temporal variability [24, 26].

Our group previously reported that a single measurement of baseline stool microbial diversity was associated with infectious risk for 34 patients during induction chemotherapy (IC) for acute myelogenous leukemia (AML) [25]. Similar to other studies of ill patients, we observed instances of rapid and profound shifts in the microbiota in our AML cohort [11, 21, 22]. Thus, herein, we sought to quantify the overall intra-patient temporal variability of the oral and stool microbiome of this cohort expanded to 59 patients. In addition, we sought to determine the consequences of microbiome temporal instability on patient outcomes and clinical factors driving intra-patient temporal variability of the microbiome during IC. We chose to study such patients because of the opportunity to characterize the microbiome prior to receipt of chemotherapy and intense antibiotic exposure (i.e., prior to severe perturbations) and the capacity to obtain dense longitudinal sampling over the course of intensive treatment due to the extended inpatient nature of IC. Moreover, AML patients are at high risk for infection during IC and such infections are generally derived from the commensal microflora [21, 23]. We hypothesized that higher microbiome intra-patient temporal variability, driven by prolonged antibiotic exposure, would be associated with poorer clinical outcomes.

## Methods

### Patient recruitment and specimen collection

Study subjects included 59 newly diagnosed adult AML patients undergoing IC at MD Anderson Cancer Center (MDACC) in Houston, TX from September 2013 to October 2014. AML patients initiating inpatient IC at MDACC were approached for study inclusion unless they had systemic infection. AML patients receiving IC at MDACC are routinely prescribed a prophylactic fluoroquinolone or cephalosporin prior to the initiation of therapy. In this study, 100% of patients received routine prophylaxis, with 64% of baseline stool, and 55% of baseline oral samples taken after the patient had already started prophylactics. AML patients over 50 years receiving IC are treated in a laminar-air flow isolation until neutrophil counts recover to >500 cells/μL or until day 28. Patients aged under 50 years are admitted for the duration of the chemotherapy (approximately 4–5 days) and then followed as an outpatient with clinic visits three times a week until neutrophil recovery or 28 days.

Buccal and fecal specimens were collected from each patient at baseline, continued approximately every 96 h as available, and stopped upon neutrophil recovery. Baseline samples were considered up to 8 days before and 24 h following IC initiation. As per availability of samples, 55 (93%) of the patients had oral samples collected before or at the same time as the initiation of chemotherapy, while 35 (59%) of the patients had stool samples collected before or at the same time as the initiation of chemotherapy. The buccal mucosa of each individual was swabbed three times on each side using a Catch-All™ Sample Collection Swab (Epicentre). Patient stool samples were either collected in a stool hat or using a BBL™ CultureSwab® (BD Diagnostics). All samples were placed in sterile 2-mL cryovials and stored immediately at −80 °C until further processing.

### 16S rRNA sequencing and data processing

Bacterial genomic DNA was extracted from buccal and stool specimens using the MO BIO PowerSoil DNA Isolation Kit (MO BIO Laboratories). The 16S rRNA V4 region was PCR amplified and sequenced on the Illumina MiSeq platform using a 2 × 250-bp paired-end protocol adapted from the Human Microbiome Project (HMP) methods [16, 27]. All samples from the same patient and site were processed and sequenced together to minimize batching issues. Amplification primers contained adapters for MiSeq sequencing and single-index barcodes resulting in PCR products that were pooled and sequenced directly. Read pairs were de-multiplexed based on barcodes and merged using USEARCH v7.0.100. 16S rRNA gene sequences were allocated to specific operational taxonomic units using a UPARSE pipeline and aligned to the V4 region within the SILVA SSURef_NR99_119 database [28]. Analysis of microbiome communities was performed in R (R Core Team 2015, version 3.2.2, http://www.R-project.org), using phyloseq [29] to calculate α- and β-diversity metrics. The Shannon Diversity Index (SDI) was used for α-diversity calculations, and weighted and unweighted UniFrac for β-diversity distances [30]. The 16S V3–V4 region HMP sequencing reads were obtained from http://hmpdacc.org/HMQCP, trimmed to match the region amplified by this study, and processed identically to AML patient samples.

### Microbiome community and statistical analyses

Intra-patient temporal variability of microbial diversity was defined as the coefficient of variation (CV) of a longitudinal collection of α-diversity values, and was calculated for each patient’s set of oral and stool samples. Higher values were indicative of more variable microbial diversity. Temporal variability in community composition, or β-diversity, of each patient was determined for the oral and stool by calculating the CV of the weighted and unweighted UniFrac distances of longitudinal samples collected from each individual per site. Again, higher values were indicative of more variable communities. Pairwise differences in temporal variability across body sites were made using Mann–Whitney U test, whereas pairwise differences among infection or response groups was performed using Student’s *t*-test with Welch’s correction. Linear correlations between CVs at different body sites were determined using Pearson’s r and *P* values generated in GraphPad Prism 6.

Heatmaps analyzing genera abundance over time among patients with increasing temporal variability were generated with the publically available pheatmap R package version 1.0.8. (http://CRAN.R-project.org/package=pheatmap), and include correlation metrics calculated with R’s cor and cor.test stats package functions. *P* values were corrected for multiple comparisons using the Benjamini and Hochberg method.

For each body habitat the population was divided into quartiles based on CV of the weighted UniFrac distance values or SDI where the first quartile was defined as stable, second and third as average, and fourth as variable as previously described [15]. To determine significant differences in genera abundance between stable, average, and variable individuals, we tested for differences between groups using non-parametric Kruskal–Wallis analysis of variance in R for genera across individuals, then corrected for the false discovery rate using the Benjamini and Hochberg method.

Multivariable regression analyses were performed using base R (R Core Team 2015, version 3.2.2, http://www.R-project.org ) and included age, antibiotic type, chemotherapy regimen, and exposure to antibiotics as covariates. Antibiotic types were subdivided into three major broad spectrum β-lactam antibiotics received by this cohort, namely, cefepime, carbapenems (primarily meropenem), and piperacillin-tazobactam. Chemotherapy regimens were subdivided into fludarabine-containing regimens, high intensity non-fludarabine-containing regimens, hypomethylators, or other. Fludarabine-containing regimens included fludarabine in combination with idarubicin and cytarabine [31], or fludarabine/idarubicin/cytarabine with G-CSF (FLAG-Ida). High intensity non-fludarabine-containing regimens were purine analog of clofarabine or cladrabine in combination with idarubicin and cytarabine. Hypomethylator-based combinations included decitabine and azacytidine [32].

### Clinical definitions

Infections were defined as microbiologically defined infections (MDIs) or clinically defined infections as described previously [25]. Subsequent infectious episodes were defined as MDIs that occurred within 90 days of cessation of longitudinal sampling. Complete remission (CR) of AML was assessed using standard definitions [33].

## Results

### AML patients undergoing IC exhibit temporal instability of the stool and oral microbiome diversity

In order to understand the intra-patient temporal variability of the microbiome among hospitalized patients with AML, we performed sequencing of the V4 region of the 16S rRNA gene via the MiSeq platform (Illumina) using the 2 × 250-bp protocol [34] on a total of 901 longitudinal samples collected twice weekly from initiation of chemotherapy until neutrophil recovery for 59 AML patients undergoing IC. Of the samples, 848 (84%, *n* = 365 stool and 483 oral) passed sequencing quality control measures for further analyses. For these samples, we obtained a total of 24,271,698 reads, for an average of 28,622 reads per sample. Patient demographics and clinical metadata can be found in Table 1.

**Table 1:** Clinical features of 59 AML patients

Characteristic	Number (%)
Demographics	
Median age in years ^a^	55 (49–68)
Male	31 (52.5)
Female	28 (47.5)
Median days on study	28 (25–35)
Median number of oral samples	8 (6–9)
Median number of stool samples	6 (4–8)
Chemotherapy	
Hypomethylators^b^	14 (23.7)
Non-fludarabine high intensity^c^	19 (32.2)
Fludarabine-containing^d^	19 (32.2)
Other^e^	7 (11.8)
Chemotherapeutic response	
Complete remission after IC	20 (33.8)
Overall response rate^f^	43 (72.8)
Infections^g^	
Microbiologically documented infection	15 (25.4)
Clinically documented infection	14 (23.7)
No infection	30 (50.8)
Antimicrobial administration	
Received treatment antibiotics^h^	53 (89.8)
Carbapenem >72 h	39 (66.1)
Piperacillin/tazobactam >72 h	14 (23.7)
Cefepime >72 h	26 (44.1)
Received prophylactic antibiotics	59 (100)
Median number of antibiotics administered	6 (4–7)
Median number of days exposed to all antibiotics^i^	28 (24–35)
Median number of days exposed to treatment antibiotics	16 (9–24)
Median number of days exposed to prophylactic antibiotics	16 (8–28)

^a^ All median values in this table have the interquartile range in parentheses

^b^ These chemotherapies included: 1) vasoroxin in combination with decitabine; 2) decitabine alone; 3) azacytidine in combination with pracinostat; 4) azacytidine in combination with quidartinib; and 5) SGI-110

^c^ These chemotherapies included: 1) CIA, 2) CLIA, 3) or CIA + sorafanib

^d^ These chemotherapies included: 1) FLAG-Ida or 2) FIA regimens

^e^Other chemotherapies included:1) omacetaxine in combination with low-dose cytarabine or 2) Clad + LDAC

^f^ Includes CR (morphologic complete remission), CRi (morphologic complete remission with incomplete bloodcount recovery), and CRp (morphologic complete remission with incomplete platelet recovery)

^g^ Specific information on microbiologically and clinically documented infections can be found in the “Methods”

^h^ Refers to any antibiotic/antimicrobial-based therapy given for suspected or proven infection, that is, not included as prophylaxis (cephalosporins or fluoroquinilones). Denoted are the three most common broad spectrum antibiotics given in the study. Note that numbers of individual antibiotics add up to >100% because some patients received more than one of the listed antimicrobials during IC

^i^ Includes prophylactic antibiotics

**Fig. 1:**
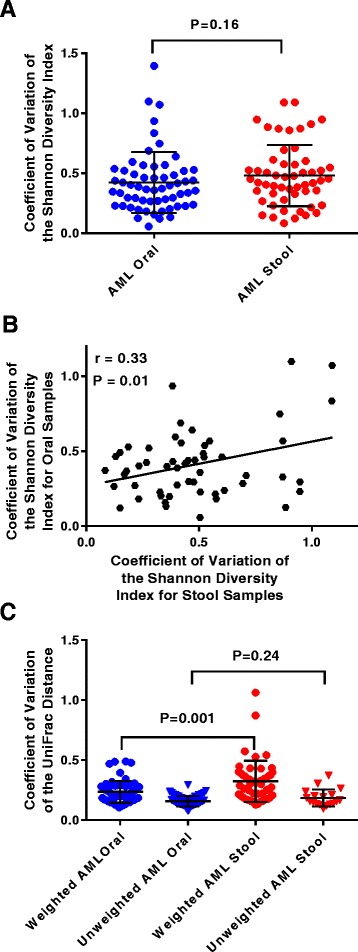
Intra-patient temporal variability in oral and stool microbiomes of hospitalized AML patients undergoing IC. **a** The oral and stool microbial α-diversity intra-patient temporal variability. Each point represents the coefficient of variation (CV) of the Shannon diversity index (SDI) for samples derived from each patient. **b** The correlation between the CV of the SDI values originating from oral and stool samples from the same patient. The Pearson’s correlation (*r*) value and *P* value from correlation analyses also are indicated. **c** The oral and stool microbial β-diversity intra-patient temporal variability using either the CV of the weighted or unweighted UniFrac distances for samples derived from each patient. In panels **a** and **c**, the *bars* represent mean ± standard deviation, and *P* values comparing the different body sites were calculated using a Mann–Whitney U-test

Currently, the majority of 16 s rRNA microbiome-based data are summarized using either numerical or index-based measurements of species richness and/or evenness within a habitat (i.e., α-diversity) or characterization of differences in microbial community composition by measuring the distance or dissimilarity between samples (i.e., β-diversity). We first sought to determine intra-patient temporal variability of α-diversity by calculating the CV of the SDI for both the oral and the stool samples for each patient. The coefficient of variation is defined as the ratio of the standard deviation to the mean; thus, a low CV would mean an individual had relatively stable species diversity over time whereas a high CV would reflect more variation. We found considerable heterogeneity in the temporal stability values of both stool (mean SDI CV 0.48 ± 0.25) and oral (mean SDI CV 0.42 ± 0.26) samples among AML patients during IC (Fig. 1a). There was no statistically significant difference in CV values between the two sites (*P =* 0.16). This finding is in contrast to previous studies performed in healthy individuals where the microbiota of oral samples have been shown to be less variable compared to stool [2, 15]. The intra-patient temporal variability of other α-diversity metrics, specifically the Chao-1 diversity index and Simpson’s diversity index, were also analyzed for the oral (mean Chao CV 0.39 ± 0.18, mean Simpson CV 0.33 ± 0.24) and stool samples (mean Chao CV 0.48 ± 0.22, mean Simpson CV 0.37 ± 0.27) of the AML cohort (Additional file 1: Figure S1a, b). Assessment of the temporal variability of α-diversity also revealed that the SDI CV of oral and stool samples from the same patients were statistically moderately correlated (*P =* 0.01, *r* = 0.33; Fig. 1b). The relationship between the two sites leads to the postulation that factors influencing temporal variability of microbial diversity in treated cancer patients may be acting on both sites concurrently. Conversely, it has been reported that the variability of one body site was not associated with the variability of other body habitats in healthy cohorts [15].

### High intra-patient temporal variability of oral and stool microbiome among AML patients is associated with increased pathogenic-associated genera abundance

Next we sought to determine the temporal variability in microbiome community structure and membership as represented by quantitative and qualitative measurements of β-diversity using weighted and unweighted UniFrac distance measurements, respectively. Here, we considered the CV of each patient’s samples per site in order to characterize the dispersion of β-diversity metrics. The mean CVs of weighted and unweighted UniFrac distances for the cohort were 0.24 ± 0.1 and 0.16 ± 0.04 for the oral samples and 0.32 ± 0.2 and 0.20 ± 0.08 for stool samples, respectively (Fig. 1c). Contrary to SDI CVs, the temporal variability of the weighted UniFrac distances between the oral and stool of patients was not significantly correlated (*P =* 0.10; Additional file 1: Figure S1d). Reports in healthy persons have observed associations between diversity and temporal stability, such as individuals with a more diverse microbiome are likely to have a more stable microbiome over time [4, 14, 15]. However, we did not find any statistically significant correlations between either the baseline or median SDI values of patients and their temporal variability as measured by the CV of the weighted UniFrac distances of their samples, suggesting microbiome structural variability does not appear to be affected by α-diversity in treated AML patients (Additional file 1: Figure S2).

**Fig. 2:**
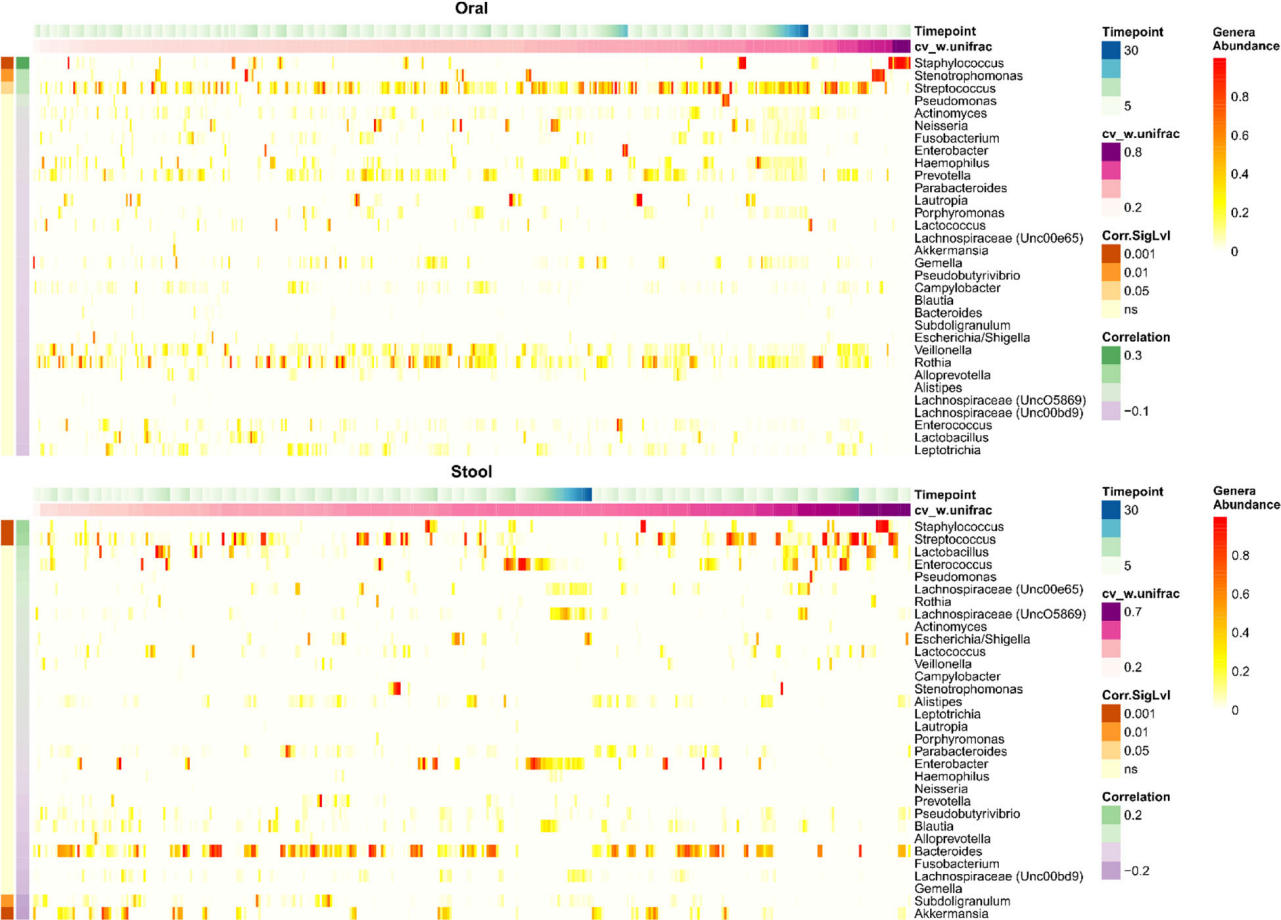
Temporal instability of microbiome community structure correlates with increasing abundance of pathogenic-associated genera over time. Heatmap of all samples and untransformed relative abundance values of indicated bacterial taxa colored *white* to *red* as denoted in the figure. Samples from each patient are clustered together and arranged by timepoint (i.e., consecutive samples) from *left* to *right*. Additionally, clusters of patient samples are organized in accordance with temporal variability as determined by the coefficient of variation of the weighted UniFrac distance (*cv_w.unifrac*) with increasing variability from *left* to *right*. Taxa are organized from *top* to *bottom* by highest positive correlation of relative abundance of genera with CV of the weighted UniFrac distance, to negative correlation as determined by Pearson’s correlation (r) values depicted in the colored inlaid figure legend. A *P* value for the correlation’s significance (*Corr.SigLvl*) was derived from the test statistic based on Pearson’s product moment correlation coefficient, corrected for multiple comparisons with the Benjamini and Hochberg method, and displayed on the plot

**Fig. 3:**
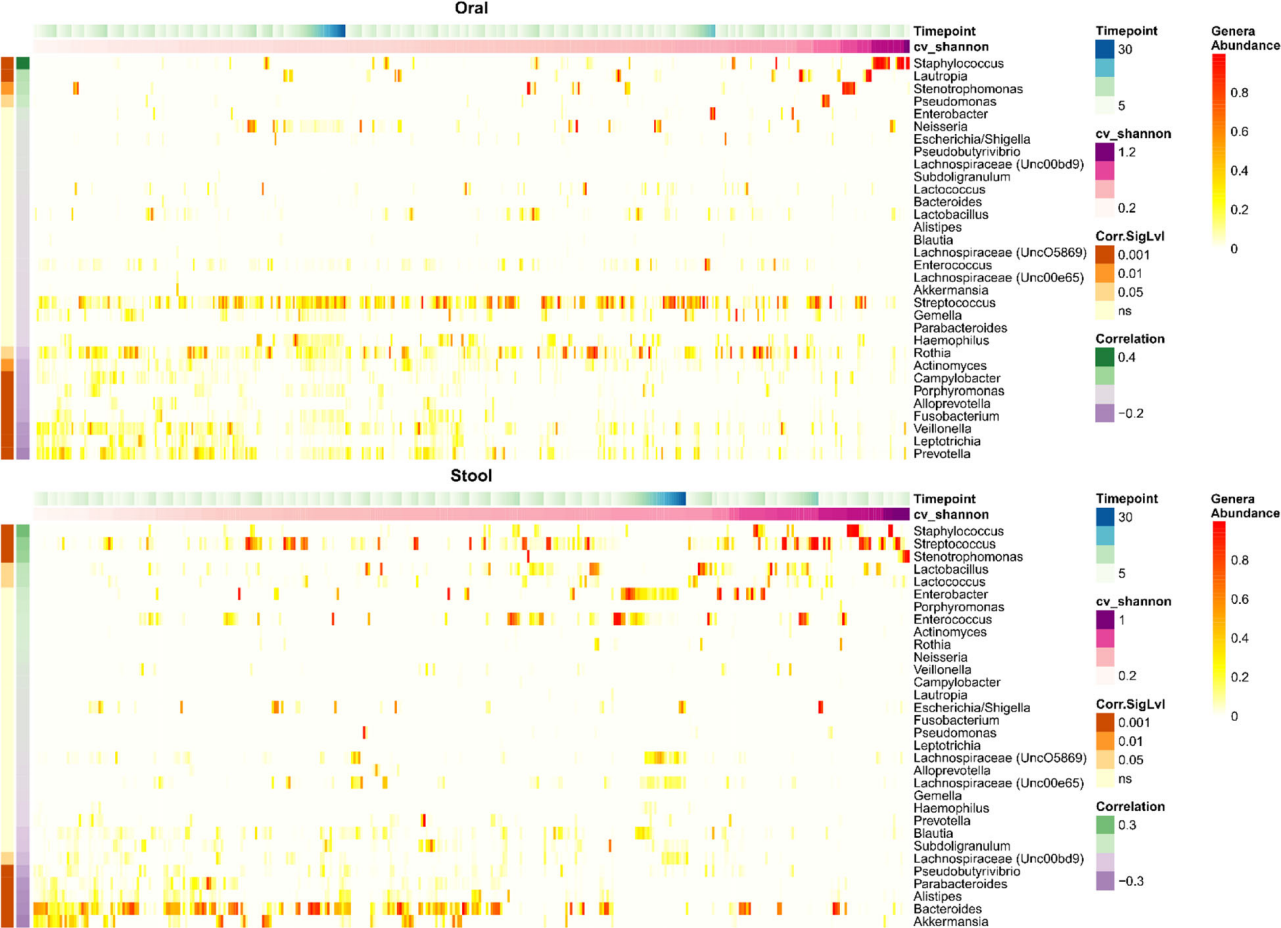
Temporal instability of microbial α-diversity correlates with increasing abundance of pathogenic-associated genera over time. This figure is arranged in the same way as Fig. 2, except temporal variability is defined using coefficient of variation of the Shannon diversity index (*cv_shannon*)

It is well known that patients in the hospital are at risk for colonization and intestinal domination by pathogenic bacteria and that a diverse microbiome provides colonization resistance against many such organisms [7, 11, 21]. Thus, to begin to investigate factors that might influence temporal instability in our cohort, we sought to test the hypothesis that temporal instability was influenced by increasing relative abundance of pathogenic-associated genera, such as *Enterococcus* and *Staphylococcus.* In order to visualize this relationship, we ranked patients and their samples by CV of weighted UniFrac from smallest to greatest (low variability to high variability of microbial community structure), and correlated this with the relative abundance of specific genera (Fig. 2). Consistent with our hypothesis, high weighted UniFrac CV values were moderately positively correlated with the relative abundance of pathogenic genera such as *Staphylococcus* (*P* < 0.001, *r* = 0.3), *Streptococcus* (*P* = 0.02, *r* = 0.2), and *Stenotrophomonas* (*P* = 0.01, *r* = 0.2) in the oral samples. Similarly, instability of community structure was statistically positively correlated with the abundance of *Staphylococcus* (*P* < 0.001, *r* = 0.2) and *Streptococcus* (*P* < 0.001, *r* = 0.2) in the stool samples. The same genera were correlated with temporal instability of microbial α-diversity (SDI CV) in oral and stool samples as well (Fig. 3). Also of note, the abundance of non-pathogenic organisms in the stool, such as *Akkermansia*, were associated with increased temporal stability of both the microbiome community structure and diversity (both stool weighted UniFrac CV and SDI CV *P* < 0.001, *r* = −0.2). Thus, our data imply that patients with a relatively high relative abundance of commensal organisms, like *Akkermansia*, maintain a more stable microbiome over the course of their hospitalization whereas higher relative abundances of pathogenic-associated bacteria, like *Staphylococcus* and *Streptococcus*, are associated with temporal instability of both the oral and gastrointestinal microbiome.

### Stabilizing and destabilizing taxa can be inferred by categorizing AML patients into stable, average, or variable microbiomes during IC

**Fig. 4:**
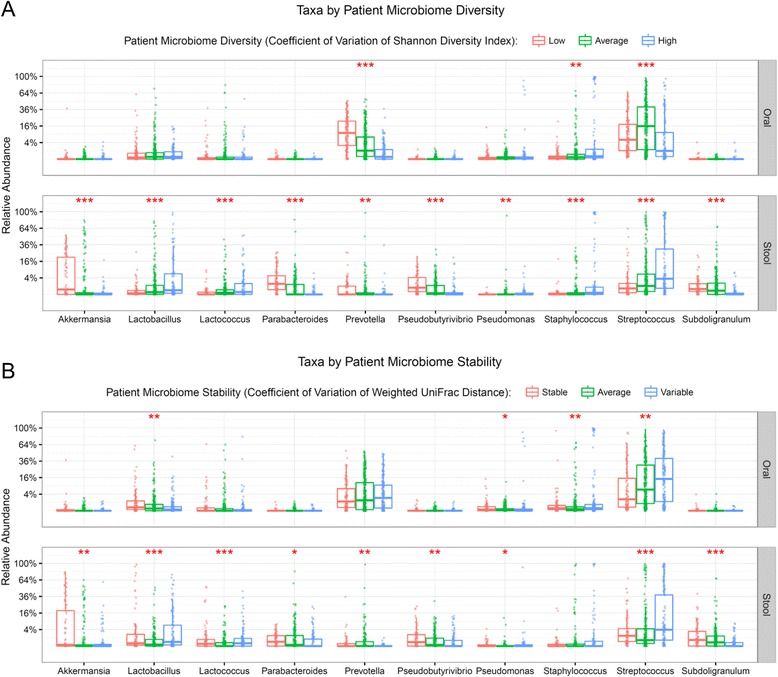
Taxonomic composition differences among different stability categories. The significant differences in relative abundances of genera between different patient stability categories based on either the coefficient of variation (CV) of the Shannon diversity index (SDI; *top two panels*) or the CV of the weighted UniFrac distances (*bottom two panels*). For each body habitat the population was divided into quartiles, where the first quartile was defined as low/stable, second and third as average, and fourth as high/variable. Differences in genera abundance across categories were determined using non-parametric Kruskal–Wallis analysis of variance, then corrected for the false discovery rate using the Benjamini and Hochberg method. *Asterisks* indicate adjusted *P* values: *P ≤ 0.05, **P ≤ 0.01, ***P ≤ 0.001, respectively. **a** Taxa by patient microbiome diversity. **b** Taxa by patient microbiome stability

We next sought to determine if we could infer stabilizing or destabilizing taxa by looking at significant differences in the relative abundance of taxa between patients with stable, average, or variable microbiomes based on their intra-patient microbial temporal stability. Patients were defined as stable, average, or variable according to their weighted UniFrac CV and SDI CV by dividing the population into quartiles with the bottom quartile (lowest CVs) being considered stable, and the top quartile (highest CVs) being considered variable. Figure 4 denotes all genera that had statistically significant abundance differences between stability categories defined by both SDI and weighted UniFrac CVs at the same body site. Pathogenic associated genera such as *Streptococcus* and *Staphylococcus* were more abundant in both oral and stool samples of individuals that were categorized as temporally variable in both the α- and β-diversity (Fig. 4). Confirming the analyses performed in Fig. 2, *Akkermensia* and *Subdilogranulum* were more abundant in the stool of stable individuals, alluding to a possible stabilizing effect of these genera. Additionally, *Pseudobutyrivibrio* was also found in greater abundance in the stool of individuals with a stable microbiome, signifying its potential importance in microbiome integrity as well. Other results for relative abundance differences in specific genera (those >1% abundance) between patient stability categories can be found in Additional file 1: Figure S3.

### Intra-patient temporal instability of microbial diversity is linked to adverse infectious outcomes during and after IC

To evaluate the clinical consequences of the observed differences in temporal stability in our cohort, we sought to determine if intra-patient temporal variability of the microbiome could be linked with adverse outcomes during and following IC. Specifically, we analyzed if the measures of temporal variability (CVs of the SDI and weighted and unweighted Unifrac distances) were correlated with infection during IC, infection in the 90 days post-IC neutrophil recovery, or response of the leukemia to chemotherapy.

**Fig. 5:**
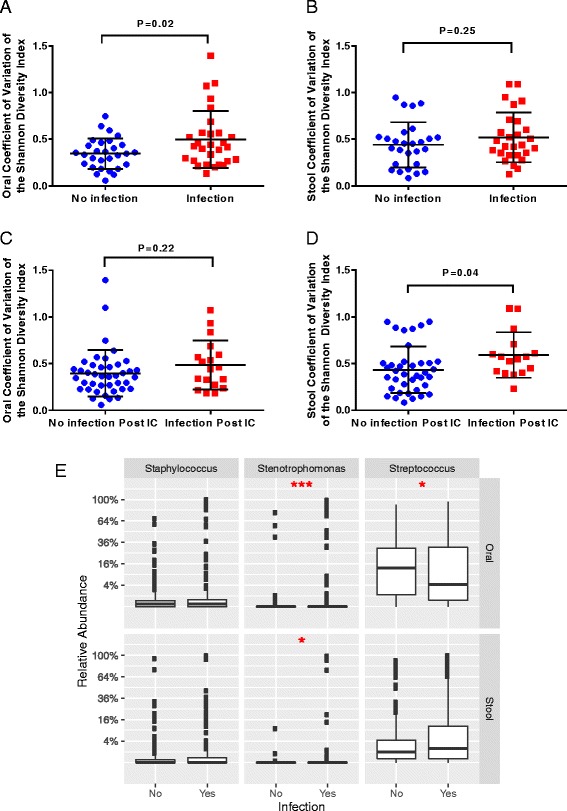
Temporal instability of microbiome α-diversity is associated with infectious outcomes during and after chemotherapy. Shown are the coefficient of variation (CV) of the Shannon diversity index (SDI) for oral (**a**) and stool (**b**) samples stratified by patients who did or did not contract an infection during the induction phase of chemotherapy before neutrophil recovery. **c**, **d** The CVs of the SDI for oral and stool samples, respectively, stratified by patients who did or did not contract an infection in the 90 days following neutrophil recovery. In all panels the *bars* represent mean ± standard deviation, and *P* values comparing SDI CV values among infectious outcomes were calculated using a two-sample *t*-test with Welch’s correction. **e** Summarized genera abundance differences between patients who did or did not contract an infection during IC. Significance was determined by individual Mann–Whitney tests for the three different genera (*P < 0.05, **P < 0.01, ***P < 0.001)

Although temporal variability of neither oral nor stool community membership (unweighted UniFrac CV) and structure (weighted UniFrac CV) was significantly correlated with infection during IC (Additional file 1: Figure S4), patients who had higher levels of oral variability based on α-diversity had significantly higher rates of infection during IC (*P =* 0.02) (Fig. 5a, b). Conversely, stool microbial α-diversity temporal variability levels, but not oral, were significantly higher during IC in patients who subsequently developed an infection within 90 days post-neutrophil recovery from IC (*P =* 0.04; Fig. 5c, d). None of the intra-patient microbiome temporal variability outputs were significantly associated with response of the leukemia to chemotherapy (Additional file 1: Figure S5). Moreover, we went on to assess if those genera found to be statistically correlated with microbial instability (e.g., *Staphylococcus*, *Streptococcus*, and *Stenotrophomonas*) were also correlated with infection. Indeed, individuals who contracted an infection during IC exhibited significantly higher relative abundances of *Streptococcus* (*P* < 0.05 for oral) and *Stenotrophomonas* (*P* < 0.001 for oral, *P* < 0.05 for stool) compared to those that did not. (Fig. 5e). These data indicate temporal variability and microbiome composition dynamics are likely important markers for infectious risk during and after IC.

### Duration of antibiotic treatment is associated with temporal instability of the oral microbiome during IC in AML patients

Due to the fact that temporal instability of the oral and gastrointestinal microbiome is associated with infectious risk among AML patients, we sought to determine clinical factors associated with microbiome temporal instability. Multivariable regression analysis was performed using clinical variables previously implicated in microbiome stability, including age, antibiotic type and duration, and chemotherapy regimen [2, 7, 35–37]. Only total days on antibiotics was statistically correlated with temporal variability of the oral microbial diversity (SDI CV *P =* 0.031), community membership (unweighted UniFrac CV *P <* 0.001), and population structure (weighted UniFrac CV *P =* 0.002) measurements (Table 2; Additional file 1: Tables S1–S3). Interestingly, none of the clinical factors analyzed were significantly correlated with intra-patient temporal instability of the stool microbiome by multivariable regression analyses (Table 2; Additional file 1: Tables S4–6). Thus, our data suggest a site-specific effect of antimicrobials on temporal variability in the AML cohort.

**Table 2:** Multivariable regression analyses of potential clinical factors associated with the intra-patient temporal instability of the oral and stool microbiomes of AML patients

	*P* value					
Variables	Oral SDI CV	Oral UUCV	Oral WUCV	Stool SDI CV	Stool UUCV	Stool WUCV
Age	0.287	0.864	0.529	0.425	0.779	0.885
Received piperacillin/tazobactam >72 h	0.475	0.208	0.175	0.507	0.215	0.973
Received cefepime >72 h	0.108	0.557	0.943	0.508	0.669	0.639
Received carbapenem >72 h	0.748	0.762	0.832	0.360	0.482	0.681
Days on all antibiotics^a^	0.031^b^	0.0001^b^	0.002^b^	0.392	0.858	0.580
Days on treatment antibiotics	0.205	0.060	0.163	0.917	0.633	0.451
Number of antibiotics received	0.089	0.380	0.874	0.357	0.724	0.167
Non-fludarabine high intensity chemotherapy	0.723	0.581	0.935	0.415	0.605	0.456
Hypomethylator-based chemotherapy	0.734	0.712	0.181	0.244	0.900	0.612

^a^ Includes prophylactic antibiotics

^b^ Significant *P* values

*UUCV* unweighted UniFrac coefficient of variation, *WUCV* weighted UniFrac coefficient of variation

### Prolonged antibiotic exposure is associated with long-term infectious outcomes among AML patients undergoing IC

**Fig. 6:**
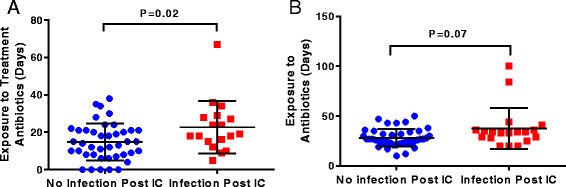
Prolonged antibiotic exposure is associated with long-term infectious complications. Illustrated are patients who did or did not contract a microbiologically determined infection in the 90 days post-neutrophil recovery and their **a** days on treatment antibiotics or **b** days on all antibiotics to include prophylaxis. In both panels the *bars* represent mean ± standard deviation, and *P* values comparing antibiotic exposure among infectious outcomes were calculated using a two-sample *t*-test with Welch’s correction

To this point we had found that total antibiotic exposure was significantly associated with microbiome temporal instability in AML patients undergoing IC, and that microbiome instability could be associated with adverse infectious outcomes. Thus, we next sought to determine if increased antibiotic exposure was associated with long-term infectious outcomes in this cohort. Indeed, individuals that developed an infection in the 90 days post-neutrophil recovery had significantly lengthier exposure to treatment antibiotics (*P =* 0.02; Fig. 6a). When exposure to all antibiotics, to include prophylactic antimicrobials, was analyzed between infection and non-infection groups post-IC, there was a trend towards increasing infection rates among patients who had received longer duration of antimicrobials, although it was not statistically significant (*P =* 0.07; Fig. 6b).

## Discussion

There is tremendous enthusiasm for using measurements and manipulation of the microbiome as a means to improve different aspects of human health. Indeed, multiple studies have shown that the microbiome can significantly impact a broad variety of pathophysiologic processes, from carcinogenesis to autoimmunity to serious infections [7, 9, 18, 24, 25, 38, 39]. As almost all of the existing datasets ascertaining longitudinal variability exist in healthy controls, the limited understanding of the variability in microbiota composition for sick patients impedes the capacity to readily translate microbiome measurements into the clinical setting. The urgent need to monitor and understand microbiome dysbiosis during critical illness has been expressed with recent initiatives such as the ICU Microbiome Project (http://americangut.org/the-icu-microbiome-project-is-there-a-better-way-to-treat-infections-than-antibiotics/), the National Microbiome Initiative (https://www.whitehouse.gov/the-press-office/2016/05/12/fact-sheet-announcing-national-microbiome-initiative), and the Center for Disease Control’s recent Broad Agency Announcement for Advanced and Innovative Solutions to Improve Public Health, which includes the request for microbiome assessment and intervention to address antibiotic resistance in both healthy individuals and in healthcare settings. Herein, we contribute to addressing this knowledge gap by analyzing the inter-patient variability of both the oral and stool microbiome for 59 AML patients using >800 samples collected over a median of 28 days, the factors driving differential variability in this cohort, and the association of inter-patient variability with clinical outcomes.

When considering how our cohort of leukemia patients compares to healthy individuals, we obtained, trimmed, and processed data from the Human Microbiome Project (HMP) [27] to match the V4 region of the 16S rRNA gene amplified by this study’s primers and processing protocols. Although a direct statistical comparison is not suitable due to differences between study methodologies, our mean SDI CV values for both oral and stool were two- to fourfold higher than both the HMP as well as a separate cohort of healthy subjects studied by Flores et al. [15, 16] (HMP mean SDI CV values were 0.2 for oral and 0.09 for stool, and mean SDI CV for stool and tongue samples for the Flores et al. data set was approximately 0.17 and 0.1, respectively), indicating that our cohort of treated AML patients had high intra-patient temporal variability of α-diversity compared to healthy subjects (Additional file 1: Figure S1c). A high level of intra-patient temporal variability found within our cohort is in concordance with observations of rapid fluctuations in microbiota composition reported in previous cohorts of intensive care unit and stem cell transplant patients [7, 11, 21].

Although the overall cohort had high levels of variability, there was a wide range, showing that many patients maintained a relatively stable microbiome despite the significant stress of AML therapy and a prolonged hospital stay. It has been previously demonstrated that specific enterotypes and the diversity of the microbiome influence the potential adverse impact of antibiotics on microbial communities [40]. In our cohort, variability was associated with the predominance of potentially pathogenic genera such as *Staphylococcus* whereas more stable microbiomes were characterized by high levels of commensals such as *Akkermansia*, *Subdilogranulum*, and *Pseudobutyrivibrio* (Figs. 2, 3, and 5). *Akkermansia* spp. have been described to be important in host metabolic homeostasis, anti-inflammatory functions, such as interleukin secretion, and promoting intestinal epithelial integrity in murine models and human colonic cell lines [38, 41]. Moreover, butyrate-producing commensal microorganisms, like *Akkermansia* and *Pseudobutyrivibrio*, are important in maintaining the health of the intestinal epithelium, which in turn provides nutrients necessary for microbiota stability [9, 38, 42]. However, *Akkermansia* spp. have also been recently associated with loss of the colonic mucus layer and compromised intestinal barrier function in mice with graft versus host disease [43]. More perplexing was the increased abundance of *Lactobacillus* and *Lactococcus* in the stool of variable individuals, as these organisms have often been associated with microbiome maintenance and mucosal integrity [39, 44–47]. These results suggest the importance of performing metagenomic shotgun sequencing in order to determine the specific species among these complex genera that are associated with maintenance and variability. Therefore, in combination with the aforementioned studies, our data appear to suggest that stable microbiomes with high levels of specific commensal microorganisms might be implicated in mechanisms protecting patients from intestinal domination and subsequent infection by pathogenic bacteria. These observations raise the question of whether fecal microbiota transplantation or targeted species repletion of patients with a microbiota dominated by pathogenic bacteria could restore intestinal homeostasis [8].

Interestingly, although antibiotic exposure is often associated with reduced stool microbial diversity [7, 35, 36], our results show total days on antibiotics was significantly correlated with temporal variability of the oral microbial diversity, but not the stool. These findings are in agreement with recent findings using generalized linear models which identified antibiotic use as a significant predictor of temporal variability of the tongue, but not the gut, in healthy subjects [15]. Given previous literature associating other clinical factors, such as the age and chemotherapy, with microbiome variability and dysbiosis, it was surprising that total antibiotic exposure to all antibiotics, including prophylaxis, was the only clinical variable tested to be statistically correlated with any measure of temporal variability [2, 37]. This suggests the very treatment applied to protect the patient appears to predispose the patient to recurrent infectious-related issues. Thus, greater efforts need to be taken towards antibiotic stewardship as well as tailoring antimicrobial treatments within the context of the microbiome.

Several limitations of our study bear mentioning. First, all of our patients had a single disease and were recruited from a single center, which means we do not know how these data represent the broader array of hospitalized patients. However, the relative homogeneity of the cohort was chosen in order to facilitate comparative analyses of the complex data generated in microbiome studies and to be able to characterize the microbiota prior to the patient becoming seriously ill. With this in mind, while the majority of samples were true baselines, a number of baseline samples, particularly fecal samples, were taken within the 24 h after initiation of chemotherapy due to sample availability. To our knowledge, there are no time-wise studies showing the effects of chemotherapy on the microbiome as the studies published to date comparing the microbiome before and after chemotherapy consider a wide range of days post-chemotherapy analyzed together [37]. Moreover, it is also known that the impact of chemotherapy on host factors is not typically observed until 7–10 days following chemotherapy initiation (e.g., neutropenia, mucositis, etc.). So, although we believe 24 h provides a reasonable window to collect baseline samples from patients urgently receiving chemotherapy, we cannot exclude the concern that chemotherapy could affect the microbiome within 24 h as this is currently unknown. Second, we used 16S rRNA analyses to determine microbial composition, limiting our classification of bacteria to the genus level. A species level analysis could be performed via shotgun metagenomic sequencing, which would also permit functional metabolomics studies that could help to elucidate mechanistic bases for our observations. However, applying such methodology to the very large number of samples in our study is currently cost prohibitive. Finally, although we had >800 microbiome measurements in our cohort, the complexity of the clinical course of our patients meant that we were limited in our ability to detect certain associations between clinical factors and microbiota variability. For example, most of our patients received various durations and combinations of antimicrobial administration that were problematic to reduce to discrete categories that could be incorporated into multivariable regression analyses. However, clinical studies of the microbiota in the acute care setting will face this same challenge which mandates large sample sizes and careful collection and analyses of both clinical and microbiome data.

## Conclusions

We have provided the largest dataset to date quantifying the longitudinal variability of the oral and stool microbiome in ill, hospitalized patients. We have identified particular bacterial taxa that are positively and negatively correlated with microbiome instability and demonstrated that high temporal variability is associated with increased rates of infectious outcomes. The characterization of microbiome temporal fluctuations described herein contribute to the first steps towards advancing microbiome-based diagnostic and therapeutic interventions that can be applicable in a wide range of ailments such as cancer, critical illness, and other immune-compromised individuals. Our previous data revealed low baseline stool α-diversity was associated with infectious risk during IC while decreases in both the oral and stool α-diversity between baseline and last samples were associated with infection post-IC. [25]. These previous findings, combined with the data herein, indicate that both microbial diversity as well as intra-patient temporal variability have infectious implications when studying acutely ill patients. Our finding of high intra-patient variability having clinical implications in these patients signifies that making conclusions based on single microbiome measurements in ill patients is likely to be problematic and suggests that statistical mechanisms that capture both the composition and the overall trajectory of a patient’s microbiota during illness will likely be required to fully integrate microbiome measurements into the clinical arena.

## Additional file

Additional file 1: Figures S1-S5 and Tables S1-S7
**Figure S1**. The temporal variability of the oral and stool microbial α-diversity of the AML and HMP cohorts. **Figure S2.** No correlation between temporal stability of community structure and bacterial diversity found among treated acute myeloid leukemia patients. **Figure S3.** Taxonomic composition differences among different stability categories. **Figure S4.** Temporal instability of microbiome community structure is not associated with infection during chemotherapy. **Figure S5.** Temporal variability of the microbiome is not associated with chemotherapeutic response. **Table S1.** Multivariable regression analyses of potential clinical factors associated with the intra-patient temporal instability of the stool as measured by the coefficient of variation of the Shannon diversity index. **Table S2.** Multivariable regression analyses of potential clinical factors associated with the intra-patient temporal instability of the stool as measured by the coefficient of variation of the unweighted Unifrac distance. **Table S3.** Multivariable regression analyses of potential clinical factors associated with the intra-patient temporal instability of the stool as measured by the coefficient of variation of the weighted Unifrac distance. **Table S4.** Multivariable regression analyses of potential clinical factors associated with the intra-patient temporal instability of oral samples as measured by the coefficient of variation of the Shannon diversity index. **Table S5.** Multivariable regression analyses of potential clinical factors associated with the intra-patient temporal instability of oral samples as measured by the coefficient of variation of the unweighted Unifrac distance. **Table S6.** Multivariable regression analyses of potential clinical factors associated with the intra-patient temporal instability of oral samples as measured by the coefficient of variation of the weighted Unifrac distance. **Table S7.** Biosample accession numbers. (PDF 1329 kb)

## Abbreviations

AML: Acute myelogenous leukemia
CR: Complete remission
CV: Coefficient of variation
HMP: Human Microbiome Project
IC: Induction chemotherapy
MDACC: MD Anderson Cancer Center
MDI: Microbiologically defined infection
SDI: Shannon diversity index.
