# Supplementary material for: Characterization of oral and gut microbiome temporal variability in hospitalized cancer patients
Source: Genome Med. 2017 Feb 28;9:21. doi: 10.1186/s13073-017-0409-1 (PMC5331640; doi:10.1186/s13073-017-0409-1)
Supplement: Additional file 1: Figures S1-S5 and Tables S1-S7 — Figure S1. The temporal variability of the oral and stool microbial α-diversity of the AML and HMP cohorts. Figure S2. No correlation between temporal stability of community structure and bacterial diversity found among treated acute myeloid leukemia patients. Figure S3. Taxonomic composition differences among different stability categories. Figure S4. Temporal instability of microbiome community structure is not associated with infection during chemotherapy. Figure S5. Temporal variability of the microbiome is not associated with chemotherapeutic response. Table S1. Multivariable regression analyses of potential clinical factors associated with the intra-patient temporal instability of the stool as measured by the coefficient of variation of the Shannon diversity index. Table S2. Multivariable regression analyses of potential clinical factors associated with the intra-patient temporal instability of the stool as measured by the coefficient of variation of the unweighted Unifrac distance. Table S3. Multivariable regression analyses of potential clinical factors associated with the intra-patient temporal instability of the stool as measured by the coefficient of variation of the weighted Unifrac distance. Table S4. Multivariable regression analyses of potential clinical factors associated with the intra-patient temporal instability of oral samples as measured by the coefficient of variation of the Shannon diversity index. Table S5. Multivariable regression analyses of potential clinical factors associated with the intra-patient temporal instability of oral samples as measured by the coefficient of variation of the unweighted Unifrac distance. Table S6. Multivariable regression analyses of potential clinical factors associated with the intra-patient temporal instability of oral samples as measured by the coefficient of variation of the weighted Unifrac distance. Table S7. Biosample accession numbers. (PDF 1329 kb) [file 13073_2017_409_MOESM1_ESM.pdf]

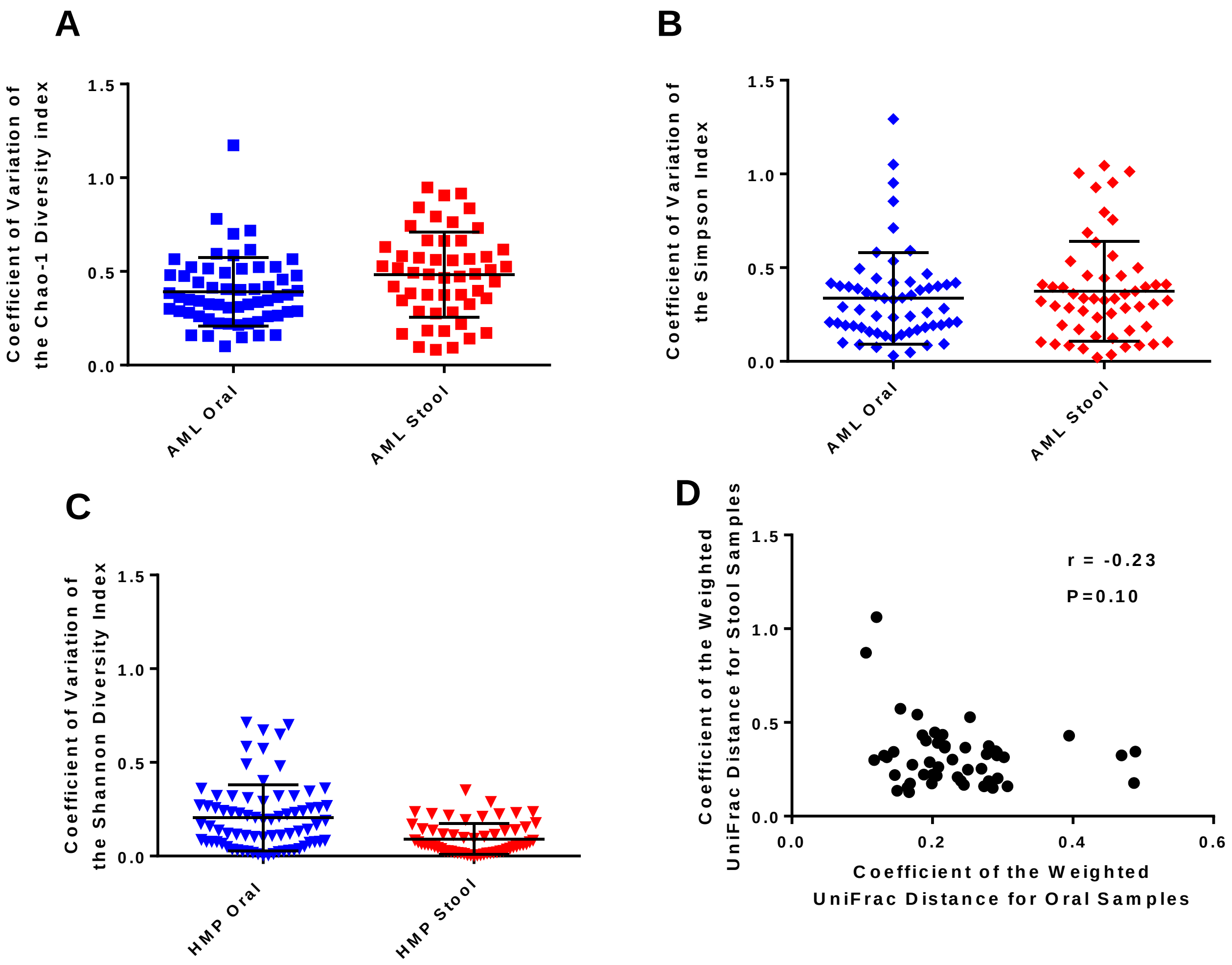

Fig. S1. The temporal variability of the oral and stool microbial α-diversity of the AML and HMP cohorts. (A) Depicted is the oral and stool microbial α-diversity intra-patient temporal variability. Each point represents the coefficient of variation (CV) of the Chao-1 diversity index for samples derived from each patient with ≥ 3 samples. (B) Same as (A) except using the Simpson index. (C) Depicted is the oral and stool microbial α-diversity intra-patient temporal variability. Each point represents the CV of the Shannon diversity index for samples derived from the HMP cohort. In panels A -C, the bars represent mean ± standard deviation values. (D) Portrayed is the correlation between the CV of the Weighted UniFrac Distances originating from oral and stool samples from the same patient. The Pearson's correlation (*r*) value and *P* value for correlation analysis also are indicated.

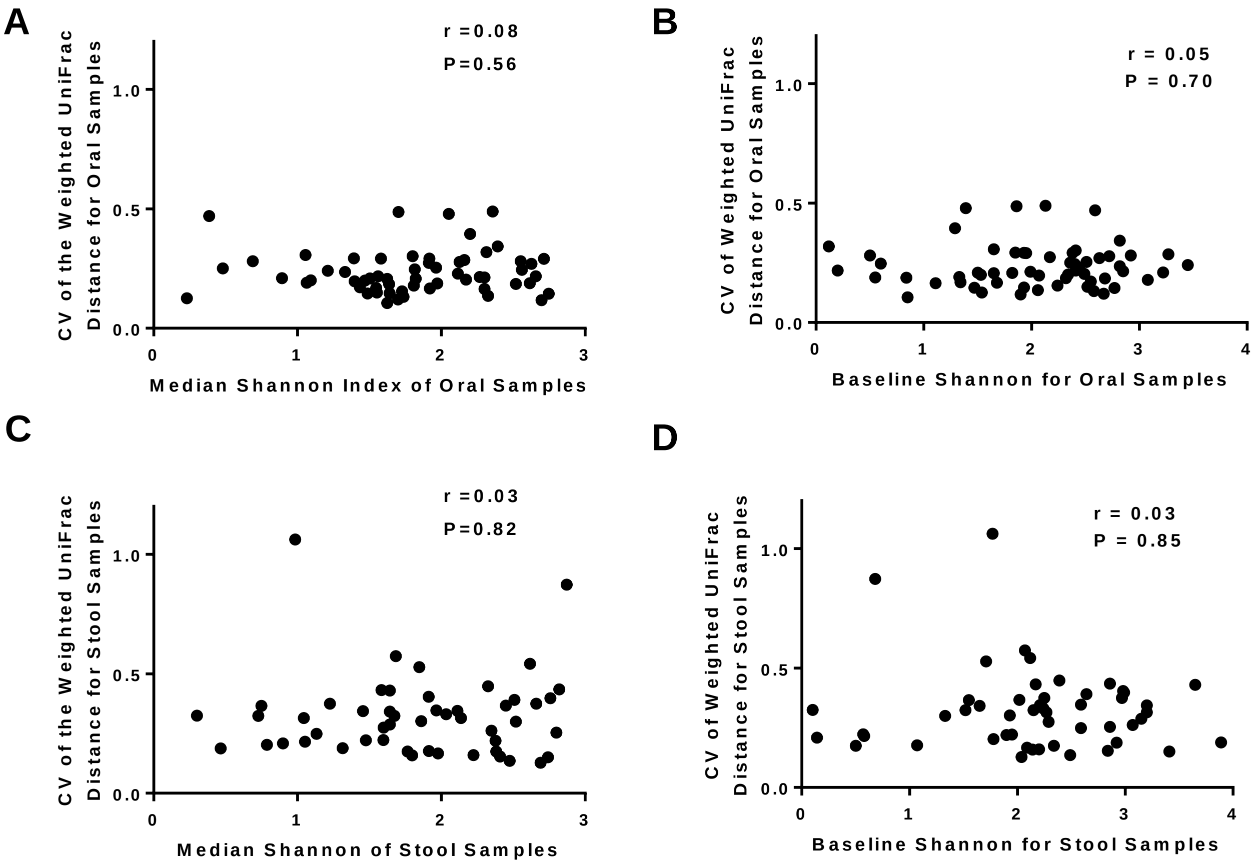

Fig. S2. No correlation between temporal stability of community structure and bacterial diversity found among treated acute myeloid leukemia patients. Portrayed is the correlation between the coefficient of variation of the weighted UniFrac distances from oral (A and B) and stool (C and D) samples from each patient and either the median Shannon index of those samples (A and C), or a patient's baseline Shannon diversity (B and D). The Pearson's correlation (r) value and *P* value for correlation analysis also are indicated.

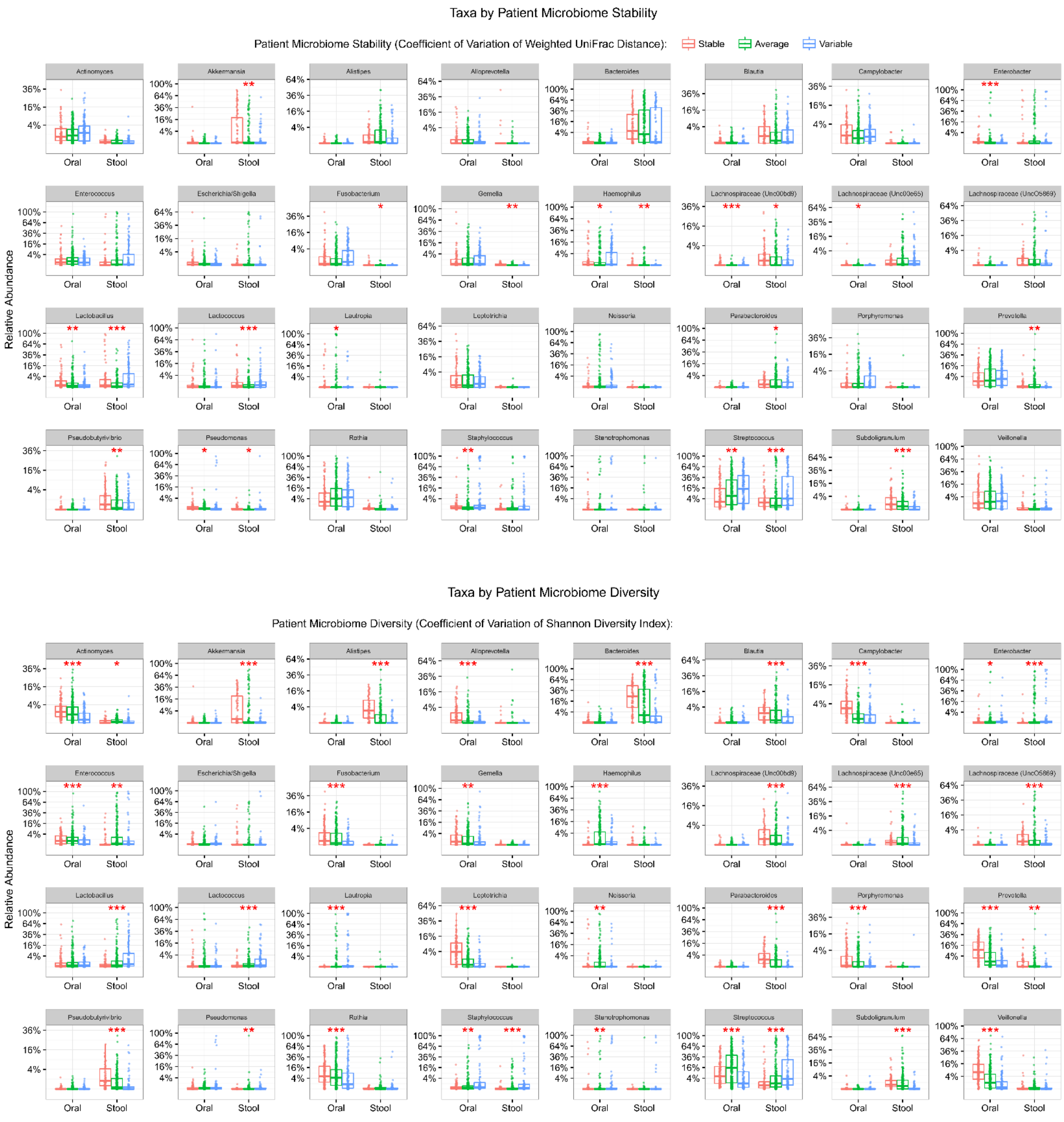

Fig. S3. Taxonomic composition differences among different stability categories. Shown are the significant differences in relative abundances of genera between different patient stability categories based on either coefficient of variation (CV) of weighted UniFrac distances (top panels) or the CV of the Shannon diversity index (SDI) (bottom panels). Differences in genera abundance across categories were determined using non-parametric Kruskal-Wallis analysis of variance, then corrected for the false discovery rate using the Benjamini & Hochberg method. Asterisk notations *, **, and *** indicate adjusted *P* values ≤0.05, 0.01, and 0.001, respectively.

Fig. S4

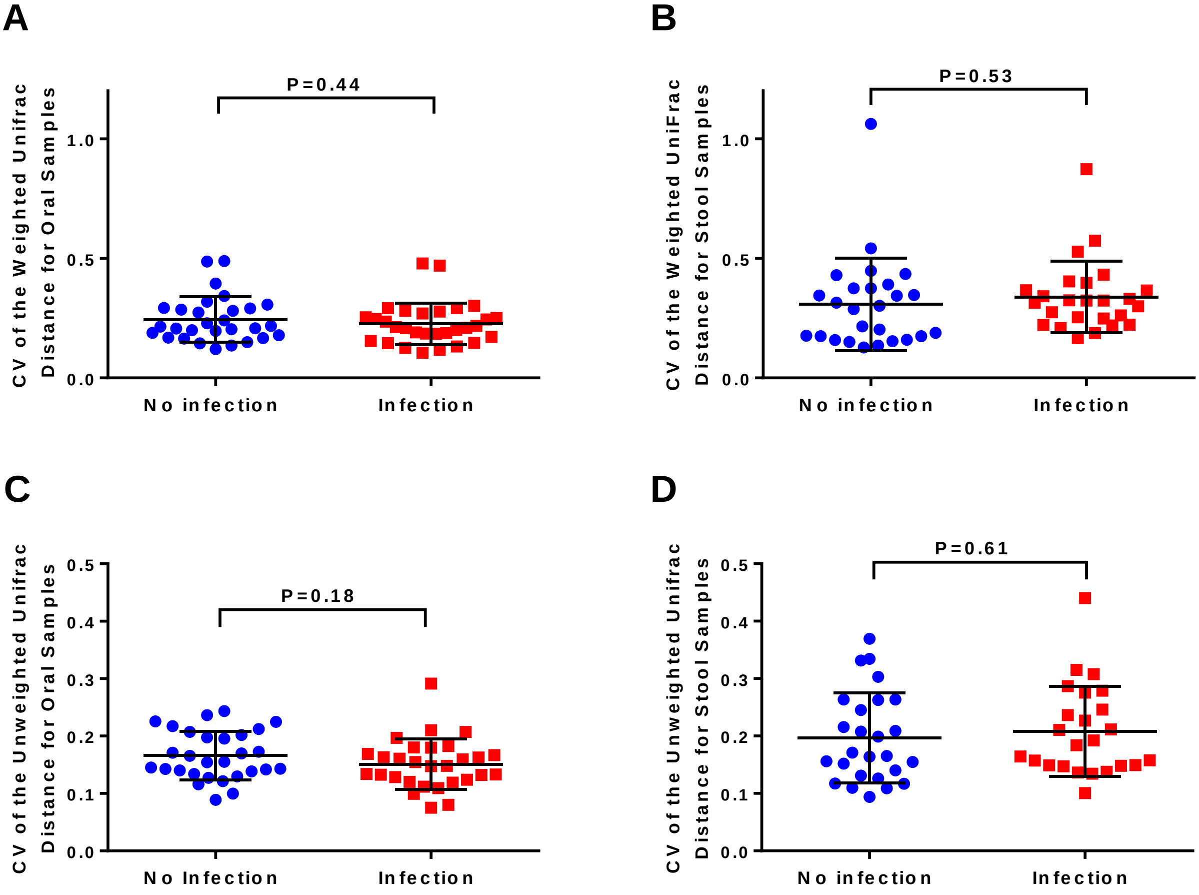

Fig. S4. Temporal instability of microbiome community structure is not associated with infection during chemotherapy. Shown are the CVs of the weighted (A and B) and unweighted (C and D) unifrac distances for oral (A and C) and stool (B and D) samples stratified by patients who did or did not contract an infection during the induction phase of chemotherapy before neutrophil recovery. In all panels the bars represent mean ± standard deviation values, and *P* values comparing CV values among infectious outcomes were calculated using a 2-sample t-test with Welch’s correction.

Fig. S5

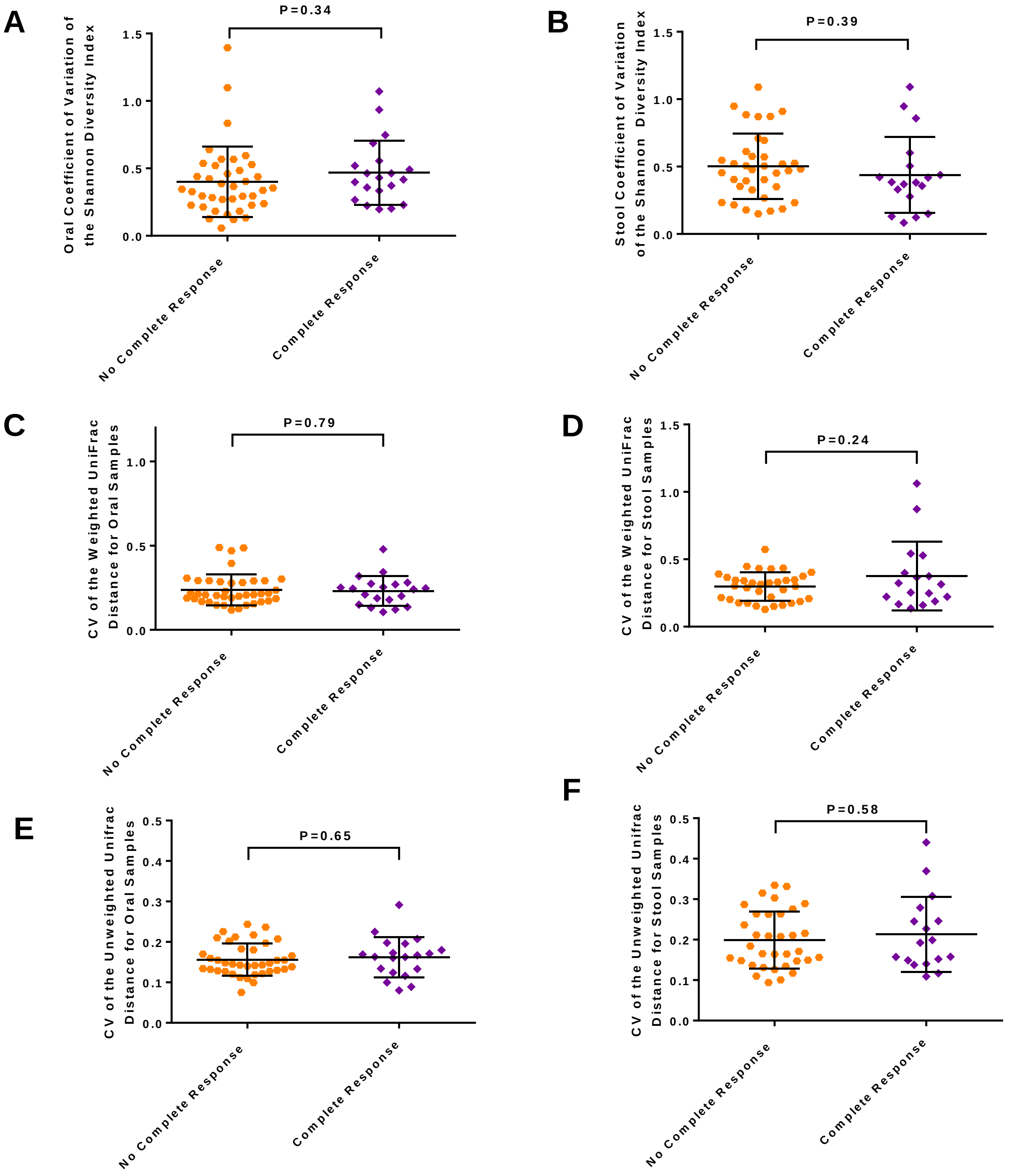

Fig. S5. Temporal variability of the microbiome is not associated with chemotherapeutic response. Shown are the CVs of the Shannon diversity index (A and B), weighted (C and D), and unweighted (E an F) unifrac distances for oral (A, C, E) and stool (B, D, F) samples stratified by patients who did or did not have a complete response to induction chemotherapy. In all panels the bars represent mean ± standard deviation values, and *P* values comparing CV values among infectious outcomes were calculated using a 2-sample t-test with Welch's correction.

Table S1. Multivariable regression analyses of potential clinical factors associated with the intra-patient temporal instability of the stool as measured by the Coefficient of Variation of the Shannon Diversity Index[1]

	Estimate	Std. Error	t value	P value
Age	-0.0030511	0.0037892	-0.805	0.425
Received Piperacillin/ Tazobactam >72hr	-0.0692387	0.1034310	-0.669	0.507
Received Cefepime >72hrs	-0.0642391	0.0961470	-0.668	0.508
Received Carbapenem >72hrs	0.0910213	0.0984667	0.924	0.360
Days on All Antibiotics[d]	0.0028492	0.0032929	0.865	0.392
Days on Treatment Antibiotics	-0.0004718	0.0045104	-0.105	0.917
Number of Antibiotics Received	0.0213972	0.0229633	0.932	0.357
Non-Fludarabine High Intensity Chemotherapy	0.0771821	0.0937103	0.824	0.415
Hypomethylator based Chemotherapy	0.1747187	0.1478379	1.182	0.244
ChemoOthers	0.0870571	0.1618069	0.538	0.593

[1] Residual standard error: 0.2507 on 43 degrees of freedom, adjusted R-squared: 0.0397 and p-value: 0.3064.

Table S2. Multivariable regression analyses of potential clinical factors associated with the intra-patient temporal instability of the stool as measured by the Coefficient of Variation of the Unweighted Unifrac Distance[1]

	Estimate	Std. Error	t value	P value
Age	-0.0003602	0.0021741	-0.283	0.7789
Received Piperacillin/ Tazobactam >72hr	0.0436241	0.0346360	1.260	0.2151
Received Cefepime >72hrs	0.0139268	0.0324944	0.430	0.6697
Received Carbapenem >72hrs	-0.0231621	0.0326590	-0.709	0.4823
Days on All Antibiotics[d]	0.0001982	0.0010993	0.180	0.8579
Days on Treatment Antibiotics	-0.0007410	0.0015409	-0.481	0.6332
Number of Antibiotics Received	0.0027624	0.0077760	0.355	0.7243
Non-Fludarabine High Intensity Chemotherapy	0.0165690	0.0317881	0.521	0.6051
Hypomethylator based Chemotherapy	-0.0062450	0.0493546	-0.127	0.8999
ChemoOthers	0.0390815	0.0533204	0.733	0.4679

[1] Residual standard error: 0.08237 on 40 degrees of freedom, adjusted R-squared: -0.1106, and p-value: 0.8784.

Table S3. Multivariable regression analyses of potential clinical factors associated with the intra-patient temporal instability of the stool as measured by the Coefficient of Variation of the Weighted Unifrac Distance[1]

	Estimate	Std. Error	t value	P value
Age	0.0004108	0.0028111	0.149	0.885
Received Piperacillin/ Tazobactam >72hr	-0.0025742	0.0764188	-0.034	0.973
Received Cefepime >72hrs	-0.0338694	0.0716937	-0.472	0.639
Received Carbapenem >72hrs	-0.0298034	0.0720568	-0.414	0.681
Days on All Antibiotics[d]	0.0013529	0.0024254	0.558	0.580
Days on Treatment Antibiotics	-0.0025904	0.0033998	-0.762	0.451
Number of Antibiotics Received	0.0241723	0.0171565	1.409	0.167
Non-Fludarabine High Intensity Chemotherapy	0.0527559	0.0701354	0.752	0.456
Hypomethylator based Chemotherapy	-0.0556192	0.1088930	-0.511	0.612
ChemoOthers	0.0886147	0.1176429	0.753	0.456

[1] Residual standard error: 0.1817 on 40 degrees of freedom, adjusted R-squared: -0.1163, and p-value: 0.8937

Table S4. Multivariable regression analyses of potential clinical factors associated with the intra-patient temporal instability of oral samples as measured by the Coefficient of Variation of the Shannon Diversity Index[1]

	Estimate	Std. Error	t value	P value
Age	0.003785	0.003515	1.077	0.2870
Received Piperacillin/ Tazobactam >72hr	-0.069843	0.097154	-0.719	0.4757
Received Cefepime >72hrs	-0.142693	0.087163	-1.637	0.1082
Received Carbapenem >72hrs	0.029032	0.089903	0.323	0.7482
Days on All Antibiotics[d]	-0.006798	0.003052	-2.227	0.0306
Days on Treatment Antibiotics	0.005466	0.004250	1.286	0.2046
Number of Antibiotics Received	0.038113	0.021953	1.736	0.0890
Non-Fludarabine High Intensity Chemotherapy	0.031210	0.087383	0.357	0.7225
Hypomethylator based Chemotherapy	-0.048031	0.140382	-0.342	0.7337
ChemoOthers	0.048609	0.140919	0.345	0.7316

[1] Residula standard error: 0.2429 on 48 degrees of freedom, adjusted R-squared: 0.08042, and p-value: 0.1661.

Table S5. Multivariable regression analyses of potential clinical factors associated with the intra-patient temporal instability of oral samples as measured by the Coefficient of Variation of the Unweighted Unifrac Distance[1]

	Estimate	Std. Error	t value	P value
Age	9.131e-01	5.338e-04	0.171	0.864908
Received Piperacillin/ Tazobactam >72hr	-1.883e-02	1.475e-02	-1.276	0.207937
Received Cefepime >72hrs	7.827e-03	1.324e-02	0.591	0.557092
Received Carbapenem >72hrs	-4.519e-03	1.365e-02	-0.305	0.761985
Days on All Antibiotics[d]	1.912e-03	4.635e-04	4.125	0.000147
Days on Treatment Antibiotics	-1.239e-03	6.445e-04	-1.920	0.060774
Number of Antibiotics Received	-2.951e-03	3.334e-03	-0.885	0.380505
Non-Fludarabine High Intensity Chemotherapy	-7.372e-03	1.327e-02	-0.556	0.581112
Hypomethylator based Chemotherapy	7.928e-03	2.132e-02	0.372	0.711619
ChemoOthers	-2.641e-02	2.140e-02	-1.234	0.223152

[1] Residual standard error: 0.03689 on 48 degrees of freedom, adjusted R-squared: 0.2682, and p-value: 0.003797

Table S6. Multivariable regression analyses of potential clinical factors associated with the intra-patient temporal instability of oral samples as measured by the Coefficient of Variation of the Weighted Unifrac Distance[1]

	Estimate	Std. Error	t value	P value
Age	-0.0007342	0.0011572	-0.634	0.52877
Received Piperacillin/ Tazobactam >72hr	-0.0439871	0.0319822	-1.375	0.17540
Received Cefepime >72hrs	-0.0020750	0.0286934	-0.072	0.94265
Received Carbapenem >72hrs	-0.0063201	0.0295955	-0.214	0.83180
Days on All Antibiotics[d]	0.0032774	0.0010047	3.262	0.00204
Days on Treatment Antibiotics	-0.0019812	0.0013992	-1.416	0.16326
Number of Antibiotics Received	-0.0011496	0.0072269	-0.159	0.87428
Non-Fludarabine High Intensity Chemotherapy	-0.0023525	0.0287658	-0.082	0.93516
Hypomethylator based Chemotherapy	0.0627605	0.0462125	1.358	0.18079
ChemoOthers	0.0362807	0.0463895	0.782	0.43800

[1] Residual standard error: 0.07998 on 48 degrees of freedom, adjusted R-squared: 0.2218, and p-value: 0.01157

Table S7. Biosample accession numbers

Accession	Sample Name
SAMN05961449	GP0001
SAMN05961450	GP0002
SAMN05961451	GP0003
SAMN05961452	GP0004
SAMN05961453	GP0005
SAMN05961454	GP0006
SAMN05961455	GP0007
SAMN05961456	GP0008
SAMN05961457	GP0009
SAMN05961458	GP0010
SAMN05961459	GP0011
SAMN05961460	GP0012
SAMN05961461	GP0013
SAMN05961462	GP0014
SAMN05961463	GP0015
SAMN05961464	GP0016
SAMN05961465	GP0017
SAMN05961466	GP0018
SAMN05961467	GP0019
SAMN05961468	GP0020
SAMN05961469	GP0021
SAMN05961470	GP0022
SAMN05961471	GP0023
SAMN05961472	GP0024
SAMN05961473	GP0025
SAMN05961474	GP0027
SAMN05961475	GP0028
SAMN05961476	GP0029
SAMN05961477	GP0030
SAMN05961478	GP0031
SAMN05961479	GP0032
SAMN05961480	GP0033
SAMN05961481	GP0034
SAMN05961482	GP0035
SAMN05961483	GP0036
SAMN05961484	GP0038
SAMN05961485	GP0039
SAMN05961486	GP0040
SAMN05961487	GP0041
SAMN05961488	GP0042
SAMN05961489	GP0043
SAMN05961490	GP0044
SAMN05961491	GP0047
SAMN05961492	GP0048
SAMN05961493	GP0049

SAMN05961494	GP0050
SAMN05961495	GP0051
SAMN05961496	GP0052
SAMN05961497	GP0053
SAMN05961498	GP0054
SAMN05961499	GP0055
SAMN05961500	GP0056
SAMN05961501	GP0057
SAMN05961502	GP0058
SAMN05961503	GP0060
SAMN05961504	GP0061
SAMN05961505	GP0062
SAMN05961506	GP0063
SAMN05961507	GP0065
SAMN05961508	GP0066
SAMN05961509	GP0068
SAMN05961510	GP0070
SAMN05961511	GP0071
SAMN05961512	GP0072
SAMN05961513	GP0073
SAMN05961514	GP0074
SAMN05961515	GP0075
SAMN05961516	GP0076
SAMN05961517	GP0077
SAMN05961518	GP0078
SAMN05961519	GP0079
SAMN05961520	GP0080
SAMN05961521	GP0082
SAMN05961522	GP0084
SAMN05961523	GP0085
SAMN05961524	GP0086
SAMN05961525	GP0088
SAMN05961526	GP0089
SAMN05961527	GP0090
SAMN05961528	GP0091
SAMN05961529	GP0092
SAMN05961530	GP0093
SAMN05961531	GP0094
SAMN05961532	GP0095
SAMN05961533	GP0096
SAMN05961534	GP0097
SAMN05961535	GP0098
SAMN05961536	GP0099
SAMN05961537	GP0100
SAMN05961538	GP0101
SAMN05961539	GP0102
SAMN05961540	GP0103

SAMN05961541	GP0104
SAMN05961542	GP0105
SAMN05961543	GP0106
SAMN05961544	GP0107
SAMN05961545	GP0108
SAMN05961546	GP0110
SAMN05961547	GP0111
SAMN05961548	GP0112
SAMN05961549	GP0113
SAMN05961550	GP0114
SAMN05961551	GP0115
SAMN05961552	GP0116
SAMN05961553	GP0117
SAMN05961554	GP0118
SAMN05961555	GP0119
SAMN05961556	GP0120
SAMN05961557	GP0121
SAMN05961558	GP0123
SAMN05961559	GP0124
SAMN05961560	GP0125
SAMN05961561	GP0126
SAMN05961562	GP0127
SAMN05961563	GP0128
SAMN05961564	GP0129
SAMN05961565	GP0130
SAMN05961566	GP0131
SAMN05961567	GP0132
SAMN05961568	GP0133
SAMN05961569	GP0134
SAMN05961570	GP0135
SAMN05961571	GP0136
SAMN05961572	GP0137
SAMN05961573	GP0138
SAMN05961574	GP0139
SAMN05961575	GP0140
SAMN05961576	GP0141
SAMN05961577	GP0142
SAMN05961578	GP0143
SAMN05961579	GP0144
SAMN05961580	GP0145
SAMN05961581	GP0146
SAMN05961582	GP0147
SAMN05961583	GP0148
SAMN05961584	GP0149
SAMN05961585	GP0150
SAMN05961586	GP0151
SAMN05961587	GP0152

SAMN05961588	GP0153
SAMN05961589	GP0154
SAMN05961590	GP0155
SAMN05961591	GP0156
SAMN05961592	GP0157
SAMN05961593	GP0158
SAMN05961594	GP0159
SAMN05961595	GP0160
SAMN05961596	GP0161
SAMN05961597	GP0162
SAMN05961598	GP0163
SAMN05961599	GP0164
SAMN05961600	GP0165
SAMN05961601	GP0166
SAMN05961602	GP0167
SAMN05961603	GP0168
SAMN05961604	GP0169
SAMN05961605	GP0170
SAMN05961606	GP0171
SAMN05961607	GP0172
SAMN05961608	GP0174
SAMN05961609	GP0176
SAMN05961610	GP0178
SAMN05961611	GP0180
SAMN05961612	GP0182
SAMN05961613	GP0183
SAMN05961614	GP0185
SAMN05961615	GP0186
SAMN05961616	GP0187
SAMN05961617	GP0189
SAMN05961618	GP0190
SAMN05961619	GP0191
SAMN05961620	GP0193
SAMN05961621	GP0197
SAMN05961622	GP0198
SAMN05961623	GP0199
SAMN05961624	GP0201
SAMN05961625	GP0203
SAMN05961626	GP0205
SAMN05961627	GP0207
SAMN05961628	GP0208
SAMN05961629	GP0209
SAMN05961630	GP0210
SAMN05961631	GP0211
SAMN05961632	GP0212
SAMN05961633	GP0214
SAMN05961634	GP0216

SAMN05961635	GP0217
SAMN05961636	GP0218
SAMN05961637	GP0219
SAMN05961638	GP0220
SAMN05961639	GP0221
SAMN05961640	GP0222
SAMN05961641	GP0223
SAMN05961642	GP0224
SAMN05961643	GP0225
SAMN05961644	GP0226
SAMN05961645	GP0227
SAMN05961646	GP0228
SAMN05961647	GP0229
SAMN05961648	GP0230
SAMN05961649	GP0231
SAMN05961650	GP0232
SAMN05961651	GP0233
SAMN05961652	GP0234
SAMN05961653	GP0235
SAMN05961654	GP0236
SAMN05961655	GP0237
SAMN05961656	GP0238
SAMN05961657	GP0239
SAMN05961658	GP0240
SAMN05961659	GP0241
SAMN05961660	GP0242
SAMN05961661	GP0243
SAMN05961662	GP0244
SAMN05961663	GP0245
SAMN05961664	GP0246
SAMN05961665	GP0247
SAMN05961666	GP0248
SAMN05961667	GP0249
SAMN05961668	GP0250
SAMN05961669	GP0251
SAMN05961670	GP0253
SAMN05961671	GP0254
SAMN05961672	GP0255
SAMN05961673	GP0257
SAMN05961674	GP0259
SAMN05961675	GP0261
SAMN05961676	GP0262
SAMN05961677	GP0263
SAMN05961678	GP0264
SAMN05961679	GP0265
SAMN05961680	GP0266
SAMN05961681	GP0267

SAMN05961682	GP0268
SAMN05961683	GP0269
SAMN05961684	GP0270
SAMN05961685	GP0271
SAMN05961686	GP0272
SAMN05961687	GP0273
SAMN05961688	GP0274
SAMN05961689	GP0275
SAMN05961690	GP0276
SAMN05961691	GP0277
SAMN05961692	GP0278
SAMN05961693	GP0279
SAMN05961694	GP0280
SAMN05961695	GP0281
SAMN05961696	GP0282
SAMN05961697	GP0283
SAMN05961698	GP0284
SAMN05961699	GP0285
SAMN05961700	GP0286
SAMN05961701	GP0287
SAMN05961702	GP0288
SAMN05961703	GP0289
SAMN05961704	GP0290
SAMN05961705	GP0291
SAMN05961706	GP0292
SAMN05961707	GP0294
SAMN05961708	GP0295
SAMN05961709	GP0296
SAMN05961710	GP0298
SAMN05961711	GP0299
SAMN05961712	GP0300
SAMN05961713	GP0301
SAMN05961714	GP0302
SAMN05961715	GP0303
SAMN05961716	GP0304
SAMN05961717	GP0305
SAMN05961718	GP0306
SAMN05961719	GP0307
SAMN05961720	GP0308
SAMN05961721	GP0309
SAMN05961722	GP0310
SAMN05961723	GP0311
SAMN05961724	GP0313
SAMN05961725	GP0314
SAMN05961726	GP0315
SAMN05961727	GP0316
SAMN05961728	GP0317

SAMN05961729	GP0318
SAMN05961730	GP0319
SAMN05961731	GP0320
SAMN05961732	GP0321
SAMN05961733	GP0322
SAMN05961734	GP0323
SAMN05961735	GP0324
SAMN05961736	GP0325
SAMN05961737	GP0327
SAMN05961738	GP0328
SAMN05961739	GP0329
SAMN05961740	GP0330
SAMN05961741	GP0331
SAMN05961742	GP0332
SAMN05961743	GP0333
SAMN05961744	GP0334
SAMN05961745	GP0335
SAMN05961746	GP0336
SAMN05961747	GP0337
SAMN05961748	GP0338
SAMN05961749	GP0339
SAMN05961750	GP0340
SAMN05961751	GP0341
SAMN05961752	GP0342
SAMN05961753	GP0343
SAMN05961754	GP0344
SAMN05961755	GP0345
SAMN05961756	GP0346
SAMN05961757	GP0347
SAMN05961758	GP0348
SAMN05961759	GP0349
SAMN05961760	GP0350
SAMN05961761	GP0351
SAMN05961762	GP0352
SAMN05961763	GP0353
SAMN05961764	GP0354
SAMN05961765	GP0355
SAMN05961766	GP0356
SAMN05961767	GP0357
SAMN05961768	GP0358
SAMN05961769	GP0359
SAMN05961770	GP0360
SAMN05961771	GP0361
SAMN05961772	GP0362
SAMN05961773	GP0363
SAMN05961774	GP0364
SAMN05961775	GP0365

SAMN05961776	GP0366
SAMN05961777	GP0367
SAMN05961778	GP0368
SAMN05961779	GP0369
SAMN05961780	GP0370
SAMN05961781	GP0371
SAMN05961782	GP0372
SAMN05961783	GP0373
SAMN05961784	GP0374
SAMN05961785	GP0375
SAMN05961786	GP0376
SAMN05961787	GP0377
SAMN05961788	GP0378
SAMN05961789	GP0379
SAMN05961790	GP0380
SAMN05961791	GP0381
SAMN05961792	GP0382
SAMN05961793	GP0383
SAMN05961794	GP0384
SAMN05961795	GP0385
SAMN05961796	GP0386
SAMN05961797	GP0387
SAMN05961798	GP0388
SAMN05961799	GP0389
SAMN05961800	GP0390
SAMN05961801	GP0391
SAMN05961802	GP0392
SAMN05961803	GP0393
SAMN05961804	GP0394
SAMN05961805	GP0395
SAMN05961806	GP0396
SAMN05961807	GP0397
SAMN05961808	GP0398
SAMN05961809	GP0399
SAMN05961810	GP0400
SAMN05961811	GP0401
SAMN05961812	GP0402
SAMN05961813	GP0403
SAMN05961814	GP0404
SAMN05961815	GP0405
SAMN05961816	GP0406
SAMN05961817	GP0407
SAMN05961818	GP0408
SAMN05961819	GP0409
SAMN05961820	GP0410
SAMN05961821	GP0411
SAMN05961822	GP0412

SAMN05961823	GP0413
SAMN05961824	GP0414
SAMN05961825	GP0415
SAMN05961826	GP0416
SAMN05961827	GP0417
SAMN05961828	GP0418
SAMN05961829	GP0419
SAMN05961830	GP0420
SAMN05961831	GP0421
SAMN05961832	GP0422
SAMN05961833	GP0423
SAMN05961834	GP0424
SAMN05961835	GP0425
SAMN05961836	GP0426
SAMN05961837	GP0427
SAMN05961838	GP0428
SAMN05961839	GP0429
SAMN05961840	GP0430
SAMN05961841	GP0431
SAMN05961842	GP0432
SAMN05961843	GP0433
SAMN05961844	GP0434
SAMN05961845	GP0435
SAMN05961846	GP0436
SAMN05961847	GP0437
SAMN05961848	GP0438
SAMN05961849	GP0439
SAMN05961850	GP0440
SAMN05961851	GP0441
SAMN05961852	GP0442
SAMN05961853	GP0443
SAMN05961854	GP0444
SAMN05961855	GP0445
SAMN05961856	GP0446
SAMN05961857	GP0447
SAMN05961858	GP0448
SAMN05961859	GP0449
SAMN05961860	GP0450
SAMN05961861	GP0451
SAMN05961862	GP0452
SAMN05961863	GP0453
SAMN05961864	GP0454
SAMN05961865	GP0455
SAMN05961866	GP0456
SAMN05961867	GP0457
SAMN05961868	GP0458
SAMN05961869	GP0459

SAMN05961870	GP0460
SAMN05961871	GP0461
SAMN05961872	GP0462
SAMN05961873	GP0463
SAMN05961874	GP0464
SAMN05961875	GP0465
SAMN05961876	GP0466
SAMN05961877	GP0467
SAMN05961878	GP0468
SAMN05961879	GP0469
SAMN05961880	GP0470
SAMN05961881	GP0471
SAMN05961882	GP0472
SAMN05961883	GP0473
SAMN05961884	GP0474
SAMN05961885	GP0475
SAMN05961886	GP0476
SAMN05961887	GP0477
SAMN05961888	GP0478
SAMN05961889	GP0479
SAMN05961890	GP0480
SAMN05961891	GP0481
SAMN05961892	GP0482
SAMN05961893	GP0483
SAMN05961894	GP0484
SAMN05961895	GP0485
SAMN05961896	GP0486
SAMN05961897	GP0487
SAMN05961898	GP0488
SAMN05961899	GP0489
SAMN05961900	GP0490
SAMN05961901	GP0491
SAMN05961902	GP0492
SAMN05961903	GP0493
SAMN05961904	GP0494
SAMN05961905	GP0495
SAMN05961906	GP0496
SAMN05961907	GP0497
SAMN05961908	GP0498
SAMN05961909	GP0499
SAMN05961910	GP0500
SAMN05961911	GP0501
SAMN05961912	GP0502
SAMN05961913	GP0503
SAMN05961914	GP0504
SAMN05961915	GP0505
SAMN05961916	GP0506

SAMN05961917	GP0507
SAMN05961918	GP0508
SAMN05961919	GP0509
SAMN05961920	GP0510
SAMN05961921	GP0511
SAMN05961922	GP0512
SAMN05961923	GP0513
SAMN05961924	GP0514
SAMN05961925	GP0515
SAMN05961926	GP0516
SAMN05961927	GP0517
SAMN05961928	GP0518
SAMN05961929	GP0519
SAMN05961930	GP0520
SAMN05961931	GP0521
SAMN05961932	GP0522
SAMN05961933	GP0523
SAMN05961934	GP0524
SAMN05961935	GP0525
SAMN05961936	GP0526
SAMN05961937	GP0527
SAMN05961938	GP0528
SAMN05961939	GP0529
SAMN05961940	GP0530
SAMN05961941	GP0531
SAMN05961942	GP0532
SAMN05961943	GP0533
SAMN05961944	GP0534
SAMN05961945	GP0535
SAMN05961946	GP0536
SAMN05961947	GP0537
SAMN05961948	GP0538
SAMN05961949	GP0539
SAMN05961950	GP0540
SAMN05961951	GP0541
SAMN05961952	GP0542
SAMN05961953	GP0543
SAMN05961954	GP0544
SAMN05961955	GP0545
SAMN05961956	GP0546
SAMN05961957	GP0547
SAMN05961958	GP0548
SAMN05961959	GP0549
SAMN05961960	GP0550
SAMN05961961	GP0551
SAMN05961962	GP0552
SAMN05961963	GP0553

SAMN05961964	GP0554
SAMN05961965	GP0555
SAMN05961966	GP0556
SAMN05961967	GP0557
SAMN05961968	GP0558
SAMN05961969	GP0559
SAMN05961970	GP0560
SAMN05961971	GP0561
SAMN05961972	GP0562
SAMN05961973	GP0563
SAMN05961974	GP0564
SAMN05961975	GP0565
SAMN05961976	GP0566
SAMN05961977	GP0567
SAMN05961978	GP0568
SAMN05961979	GP0569
SAMN05961980	GP0570
SAMN05961981	GP0571
SAMN05961982	GP0572
SAMN05961983	GP0573
SAMN05961984	GP0574
SAMN05961985	GP0575
SAMN05961986	GP0576
SAMN05961987	GP0577
SAMN05961988	GP0578
SAMN05961989	GP0579
SAMN05961990	GP0580
SAMN05961991	GP0581
SAMN05961992	GP0582
SAMN05961993	GP0583
SAMN05961994	GP0584
SAMN05961995	GP0585
SAMN05961996	GP0586
SAMN05961997	GP0587
SAMN05961998	GP0588
SAMN05961999	GP0589
SAMN05962000	GP0590
SAMN05962001	GP0591
SAMN05962002	GP0592
SAMN05962003	GP0593
SAMN05962004	GP0594
SAMN05962005	GP0595
SAMN05962006	GP0596
SAMN05962007	GP0597
SAMN05962008	GP0598
SAMN05962009	GP0599
SAMN05962010	GP0600

SAMN05962011	GP0601
SAMN05962012	GP0602
SAMN05962013	GP0603
SAMN05962014	GP0604
SAMN05962015	GP0605
SAMN05962016	GP0606
SAMN05962017	GP0607
SAMN05962018	GP0608
SAMN05962019	GP0609
SAMN05962020	GP0610
SAMN05962021	GP0611
SAMN05962022	GP0612
SAMN05962023	GP0613
SAMN05962024	GP0614
SAMN05962025	GP0615
SAMN05962026	GP0616
SAMN05962027	GP0617
SAMN05962028	GP0618
SAMN05962029	GP0619
SAMN05962030	GP0620
SAMN05962031	GP0621
SAMN05962032	GP0622
SAMN05962033	GP0623
SAMN05962034	GP0624
SAMN05962035	GP0625
SAMN05962036	GP0626
SAMN05962037	GP0627
SAMN05962038	GP0628
SAMN05962039	GP0629
SAMN05962040	GP0630
SAMN05962041	GP0631
SAMN05962042	GP0632
SAMN05962043	GP0633
SAMN05962044	GP0634
SAMN05962045	GP0635
SAMN05962046	GP0636
SAMN05962047	GP0637
SAMN05962048	GP0638
SAMN05962049	GP0639
SAMN05962050	GP0640
SAMN05962051	GP0641
SAMN05962052	GP0642
SAMN05962053	GP0643
SAMN05962054	GP0644
SAMN05962055	GP0645
SAMN05962056	GP0646
SAMN05962057	GP0647

SAMN05962058	GP0648
SAMN05962059	GP0649
SAMN05962060	GP0650
SAMN05962061	GP0651
SAMN05962062	GP0652
SAMN05962063	GP0654
SAMN05962064	GP0655
SAMN05962065	GP0656
SAMN05962066	GP0657
SAMN05962067	GP0658
SAMN05962068	GP0659
SAMN05962069	GP0660
SAMN05962070	GP0661
SAMN05962071	GP0662
SAMN05962072	GP0663
SAMN05962073	GP0664
SAMN05962074	GP0665
SAMN05962075	GP0666
SAMN05962076	GP0667
SAMN05962077	GP0668
SAMN05962078	GP0669
SAMN05962079	GP0671
SAMN05962080	GP0672
SAMN05962081	GP0673
SAMN05962082	GP0674
SAMN05962083	GP0675
SAMN05962084	GP0676
SAMN05962085	GP0677
SAMN05962086	GP0678
SAMN05962087	GP0679
SAMN05962088	GP0680
SAMN05962089	GP0681
SAMN05962090	GP0682
SAMN05962091	GP0683
SAMN05962092	GP0684
SAMN05962093	GP0685
SAMN05962094	GP0686
SAMN05962095	GP0687
SAMN05962096	GP0688
SAMN05962097	GP0689
SAMN05962098	GP0690
SAMN05962099	GP0691
SAMN05962100	GP0692
SAMN05962101	GP0693
SAMN05962102	GP0694
SAMN05962103	GP0695
SAMN05962104	GP0696

SAMN05962105	GP0697
SAMN05962106	GP0698
SAMN05962107	GP0699
SAMN05962108	GP0700
SAMN05962109	GP0701
SAMN05962110	GP0702
SAMN05962111	GP0703
SAMN05962112	GP0704
SAMN05962113	GP0705
SAMN05962114	GP0706
SAMN05962115	GP0707
SAMN05962116	GP0708
SAMN05962117	GP0709
SAMN05962118	GP0710
SAMN05962119	GP0711
SAMN05962120	GP0712
SAMN05962121	GP0713
SAMN05962122	GP0714
SAMN05962123	GP0715
SAMN05962124	GP0716
SAMN05962125	GP0717
SAMN05962126	GP0718
SAMN05962127	GP0719
SAMN05962128	GP0720
SAMN05962129	GP0721
SAMN05962130	GP0722
SAMN05962131	GP0723
SAMN05962132	GP0724
SAMN05962133	GP0725
SAMN05962134	GP0726
SAMN05962135	GP0727
SAMN05962136	GP0728
SAMN05962137	GP0729
SAMN05962138	GP0730
SAMN05962139	GP0731
SAMN05962140	GP0732
SAMN05962141	GP0733
SAMN05962142	GP0734
SAMN05962143	GP0735
SAMN05962144	GP0736
SAMN05962145	GP0737
SAMN05962146	GP0738
SAMN05962147	GP0739
SAMN05962148	GP0740
SAMN05962149	GP0741
SAMN05962150	GP0742
SAMN05962151	GP0743

SAMN05962152	GP0744
SAMN05962153	GP0745
SAMN05962154	GP0746
SAMN05962155	GP0747
SAMN05962156	GP0748
SAMN05962157	GP0749
SAMN05962158	GP0750
SAMN05962159	GP0751
SAMN05962160	GP0752
SAMN05962161	GP0753
SAMN05962162	GP0754
SAMN05962163	GP0755
SAMN05962164	GP0756
SAMN05962165	GP0758
SAMN05962166	GP0759
SAMN05962167	GP0760
SAMN05962168	GP0762
SAMN05962169	GP0765
SAMN05962170	GP0766
SAMN05962171	GP0767
SAMN05962172	GP0768
SAMN05962173	GP0769
SAMN05962174	GP0770
SAMN05962175	GP0771
SAMN05962176	GP0772
SAMN05962177	GP0773
SAMN05962178	GP0775
SAMN05962179	GP0776
SAMN05962180	GP0777
SAMN05962181	GP0778
SAMN05962182	GP0779
SAMN05962183	GP0780
SAMN05962184	GP0781
SAMN05962185	GP0782
SAMN05962186	GP0783
SAMN05962187	GP0784
SAMN05962188	GP0785
SAMN05962189	GP0786
SAMN05962190	GP0787
SAMN05962191	GP0788
SAMN05962192	GP0790
SAMN05962193	GP0792
SAMN05962194	GP0793
SAMN05962195	GP0794
SAMN05962196	GP0795
SAMN05962197	GP0796
SAMN05962198	GP0797

SAMN05962199	GP0798
SAMN05962200	GP0799
SAMN05962201	GP0800
SAMN05962202	GP0801
SAMN05962203	GP0802
SAMN05962204	GP0803
SAMN05962205	GP0804
SAMN05962206	GP0805
SAMN05962207	GP0806
SAMN05962208	GP0807
SAMN05962209	GP0808
SAMN05962210	GP0809
SAMN05962211	GP0810
SAMN05962212	GP0811
SAMN05962213	GP0812
SAMN05962214	GP0813
SAMN05962215	GP0814
SAMN05962216	GP0815
SAMN05962217	GP0816
SAMN05962218	GP0817
SAMN05962219	GP0818
SAMN05962220	GP0819
SAMN05962221	GP0820
SAMN05962222	GP0821
SAMN05962223	GP0822
SAMN05962224	GP0823
SAMN05962225	GP0824
SAMN05962226	GP0825
SAMN05962227	GP0826
SAMN05962228	GP0827
SAMN05962229	GP0829
SAMN05962230	GP0830
SAMN05962231	GP0831
SAMN05962232	GP0832
SAMN05962233	GP0833
SAMN05962234	GP0834
SAMN05962235	GP0835
SAMN05962236	GP0836
SAMN05962237	GP0837
SAMN05962238	GP0838
SAMN05962239	GP0839
SAMN05962240	GP0840
SAMN05962241	GP0841
SAMN05962242	GP0842
SAMN05962243	GP0843
SAMN05962244	GP0844
SAMN05962245	GP0845

SAMN05962246	GP0846
SAMN05962247	GP0847
SAMN05962248	GP0848
SAMN05962249	GP0850
SAMN05962250	GP0851
SAMN05962251	GP0852
SAMN05962252	GP0853
SAMN05962253	GP0854
SAMN05962254	GP0855
SAMN05962255	GP0856
SAMN05962256	GP0857
SAMN05962257	GP0858
SAMN05962258	GP0859
SAMN05962259	GP0860
SAMN05962260	GP0861
SAMN05962261	GP0862
SAMN05962262	GP0863
SAMN05962263	GP0864
SAMN05962264	GP0865
SAMN05962265	GP0866
SAMN05962266	GP0867
SAMN05962267	GP0868
SAMN05962268	GP0869
SAMN05962269	GP0870
SAMN05962270	GP0871
SAMN05962271	GP0872
SAMN05962272	GP0873
SAMN05962273	GP0874
SAMN05962274	GP0876
SAMN05962275	GP0877
SAMN05962276	GP0878
SAMN05962277	GP0879
SAMN05962278	GP0880
SAMN05962279	GP0881
SAMN05962280	GP0882
SAMN05962281	GP0883
SAMN05962282	GP0884
SAMN05962283	GP0885
SAMN05962284	GP0886
SAMN05962285	GP0887
SAMN05962286	GP0888
SAMN05962287	GP0889
SAMN05962288	GP0891
SAMN05962289	GP0892
SAMN05962290	GP0893
SAMN05962291	GP0894
SAMN05962292	GP0895

SAMN05962293	GP0896
SAMN05962294	GP0897
SAMN05962295	GP0898
SAMN05962296	GP0899
SAMN05962297	GP0900
SAMN05962298	GP0901
